# High activity and high functional connectivity are mutually exclusive in resting state zebrafish and human brains

**DOI:** 10.1186/s12915-022-01286-3

**Published:** 2022-04-11

**Authors:** Mahdi Zarei, Dan Xie, Fei Jiang, Adil Bagirov, Bo Huang, Ashish Raj, Srikantan Nagarajan, Su Guo

**Affiliations:** 1 Department of Bioengineering and Therapeutic Sciences, Kavli Institute for Fundamental Neuroscience, University of California, San Francisco, USA; 2 Department of Pharmaceutical Chemistry, University of California, San Francisco, USA; 3 Department of Epidemiology and Biostatistics, University of California, San Francisco, USA; 4 Faculty of Science and Technology, Federation University Australia, Ballarat, Victoria Australia; 5 Chan Zuckerberg Biohub, San Francisco, CA 94143 USA; 6 Department of Radiology and Biomedical Imaging, University of California, San Francisco, USA; 7 Programs in Human Genetics and Biological Sciences, Kavli Institute for Fundamental Neuroscience, University of California, San Francisco, USA

**Keywords:** Whole brain recording at cellular resolution, Activity connectivity relationship, Anatomical and functional architecture of the brain, Functional connectome, Spontaneous activity, Selective-plane illumination microscopy (SPIM), Light sheet microscopy, Optimal thresholding values, Intrinsic brain network property, Machine learning

## Abstract

**Background:**

The structural connectivity of neurons in the brain allows active neurons to impact the physiology of target neuron types with which they are functionally connected. While the structural connectome is at the basis of functional connectome, it is the functional connectivity measured through correlations between time series of individual neurophysiological events that underlies behavioral and mental states. However, in light of the diverse neuronal cell types populating the brain and their unique connectivity properties, both neuronal activity and functional connectivity are heterogeneous across the brain, and the nature of their relationship is not clear. Here, we employ brain-wide calcium imaging at cellular resolution in larval zebrafish to understand the principles of resting state functional connectivity.

**Results:**

We recorded the spontaneous activity of >12,000 neurons in the awake resting state forebrain. By classifying their activity (i.e., variances of ΔF/F across time) and functional connectivity into three levels (high, medium, low), we find that highly active neurons have low functional connections and highly connected neurons are of low activity. This finding holds true when neuronal activity and functional connectivity data are classified into five instead of three levels, and in whole brain spontaneous activity datasets. Moreover, such activity-connectivity relationship is not observed in randomly shuffled, noise-added, or simulated datasets, suggesting that it reflects an intrinsic brain network property. Intriguingly, deploying the same analytical tools on functional magnetic resonance imaging (fMRI) data from the resting state human brain, we uncover a similar relationship between activity (signal variance over time) and functional connectivity, that is, regions of high activity are non-overlapping with those of high connectivity.

**Conclusions:**

We found a mutually exclusive relationship between high activity (signal variance over time) and high functional connectivity of neurons in zebrafish and human brains. These findings reveal a previously unknown and evolutionarily conserved brain organizational principle, which has implications for understanding disease states and designing artificial neuronal networks.

**Supplementary Information:**

The online version contains supplementary material available at 10.1186/s12915-022-01286-3.

## Background

The structure of the brain spans dimensions that have many orders of magnitudes apart, from molecules, synapses, and cells to meso- and macro-scale brain systems. Such extraordinary architecture serves not only to process sensory information that guides motor behaviors, but also to generate and maintain internal states (e.g., emotional, motivational, and cognitive states) that can critically influence an organism’s response to environment.

Diverse approaches have been employed to understand the brain’s architecture at both anatomical and functional levels across multiple scales in invertebrate and vertebrate organisms [1–9]. Viral tracing and MRI/fMRI studies provide meso- to macro-scale descriptions of connectivity in mammalian brains [10–12]. A single-cell resolution connectome of *C. elegans*’ 302 neurons is constructed at the nanometer scale using electron microscopy (EM) [1], but the application of EM to more complex brains requires enormous time and resources, making it best suited for small parts of brain tissues from one or few individuals [13–16].

While structural connectome is foundational to functional connectome, it is the functional connectome that underlies behavioral and mental states. One effective way to gain insights into brain’s functional architecture is to record spontaneous neuronal activities brain-wide and analyze their activity and functional connectivity. Functional connectivity measures correlations between time series of individual neurophysiological events [17]. Such connectivity may be direct, indirect through a subnetwork [18], or via wireless neuro-modulatory communications [19]. Brain-wide functional connectivity studies have been mainly carried out in humans using blood-oxygen-level-dependent (BOLD) fMRI [7] and MEG/EEG data [20].

Recent technological advancements in neural activity reporters [21] and fast in vivo imaging technologies [22–24] have made it possible to record whole brain activity at cellular resolution in larval zebrafish [3, 25, 26], a vertebrate model organism with relatively small and transparent brains. An elegant body of work has uncovered brain-wide dynamics underlying sensorimotor behaviors [3, 27–32]. Studies of spontaneous neuronal activities in zebrafish however have been few. Nevertheless, these studies, mostly focused on the larval optic tectum, have revealed that spontaneous activity represents “preferred” network states with propagating neuronal avalanches [33, 34]. Spontaneous activity can be reorganized over development [35] and reflects a spatial structure independent of and activate-able by visual inputs [36].

The dynamicity of activity and functional connectivity patterns in the resting state brain have long fascinated system neuroscientists [37]. The resting state brain activity refers to spontaneous activity without deliberately given stimuli. Such activity shows relatively consistent distributed patterns and can be used to characterize network dynamics without needing an explicit task to drive brain activity. Analyses of cross-correlation between activity in different brain regions demonstrate that resting state networks (RSNs) [38] and default mode networks (DMNs) [39] reflect to a considerable extent the anatomical connectivity between the regions in a network. Such intrinsic activity dynamics is shown to be disrupted in neuropsychiatric disorders [40].

In this study, we exploit the resting-state brain activity data to understand how the activity of a neuron (or neuronal population) might predict the degree of its functional connections. Since neuronal activity is an essential drive that underlies functional connectivity, we hypothesize that neurons with high activity will likely have high functional connectivity. To test this hypothesis, we applied selective-plane illumination microscopy (SPIM) [22, 41] to image individual neuron’s spontaneous activity across the forebrain of transgenic larval zebrafish expressing nuclear-targeted GCAMP6s. The vertebrate forebrain shares considerable homology in developmental ontogeny and gene expression domains and is functionally involved in sensory, emotional, and cognitive processing [42–45]. In a 6-day-old larval zebrafish, the forebrain is spontaneously active with strong local correlations and relatively reduced long-range correlations with the mid- and hindbrain areas [26, 29]. It remains unclear how such spontaneous activity in the forebrain informs the underlying functional architecture. Employing image processing methods to detect individual neurons, we obtained time-dependent activity data for more than 12K neurons per individual forebrain. Through image registration to a brain atlas [46], we assigned anatomical labels to each neuron. We established methods to identify optimal thresholding values, at which the functional connectivity was computed. By further classifying individual neurons into three activity and connectivity groups (high, medium, and low), we uncovered a surprising complementary distribution of highly active vs. highly connected neurons. Moreover, we extended such analytical methods to zebrafish whole brain and fMRI datasets from the resting state human brain. Similar results like that of the zebrafish forebrain were obtained. Similar results were also obtained using different methodologies (e.g., different numbers of k-means clusters, thresholding vs non-thresholding in the analysis of functional connectivity). Furthermore, shuffled, noise-added, or simulated datasets showed different patterns of activity-connectivity relationship, suggesting that our observation reflects an intrinsic brain network property. Together, these findings reveal a mutually exclusive relationship between high activity (signal variance over time) and high functional connectivity. Its plausible cause and implications are discussed in the “Discussion” section below.

## Results

### Light-sheet imaging and image processing generate large-scale single neuron activity data across the larval zebrafish forebrain

Using a light-sheet imaging system custom constructed based on the iSPIM design [41], we recorded the spontaneous activity of neurons in the larval zebrafish forebrain under awake resting state. For each individual, calcium imaging data were collected at ~ 2 volumes per second (26 Z planes with 4-μm interval per volume) for ~15 min (*n*=9), and acquired images were processed via an image processing pipeline, resulting in a set of multi-dimensional data (Additional file 1 A-B, Videos S1). The pan-neuronally expressed calcium indicator GCAMP6s fused to the histone H2B protein was localized to the cell nuclei. Using this feature, we segmented the brain into ROIs (region-of-interest): Each ROI is an individual neuron (Additional file 1 C). Neuronal activity as reflected by ΔF/F was calculated using time-dependent baseline estimation procedure as previously described [47].

In any calcium imaging experiment, fluorescent signal changes as a measure of neuronal activation are often plagued with noises, either from the instrument or from baseline fluctuations. To differentiate genuine neuronal activity-related peaks from such background noises, we applied a method based on the Bayesian inference of two-dimensional distribution of adjacent ΔF/F values [33]. This enabled us to obtain “ultra-cleaned” data at 99% confidence levels (Additional file 1 D). Together, these experiments generate large-scale single neuron activity data across a healthy group of individuals at the awake resting state (Additional file 1 E).

### Brain registration enables comparison of anatomically identifiable neuronal activity patterns across different individual larval zebrafish

Since individuals differ in morphology, position orientation under the imaging microscope, and GCAMP signal intensity, it is difficult to directly compare their brain activity data even though such data are acquired under identical conditions to the experimenter’s knowledge. In order to compare data across individuals, we registered the imaging stacks to the Z-brain atlas [46]. The iSPIM imaging stacks, which are acquired at a 45-degree angle to the anteroposterior axis, however, cannot be directly registered to the Z-brain template, due to (1) a significant mismatch between the image directions of our stacks and the Z-brain template and (2) a significant difference between the volumes of interest (our highly sampled forebrain vs. the whole brain). To address this problem, we created an intermediate reference brain from the Z-brain template, by resampling the forebrain region in the direction and pixel sizes that are comparable to those of the iSPIM stacks (Additional file 2 A-B). For each individual, a densely sampled Z-stack (with 1-μm interval) was collected and used for registration to the intermediate reference brain using computational morphometry toolkit (CMTK) [48]. An example of pre- and post-registration images were shown in Additional file 2 C.

We assigned the 294 anatomical masks in the Z-brain template to the registered iSPIM stacks by reformatting the coordinates for each detected neuron according to the registered frame. This process enabled us to identify anatomical labels for each neuron and the brain regions covered by our imaging volumes (Additional file 3). Taken together, these analyses generate anatomically identifiable neuronal activity data that can be compared across individuals.

### Visualization of neuronal activity landscape at single-cell resolution in the larval zebrafish forebrain

**Fig. 1:**
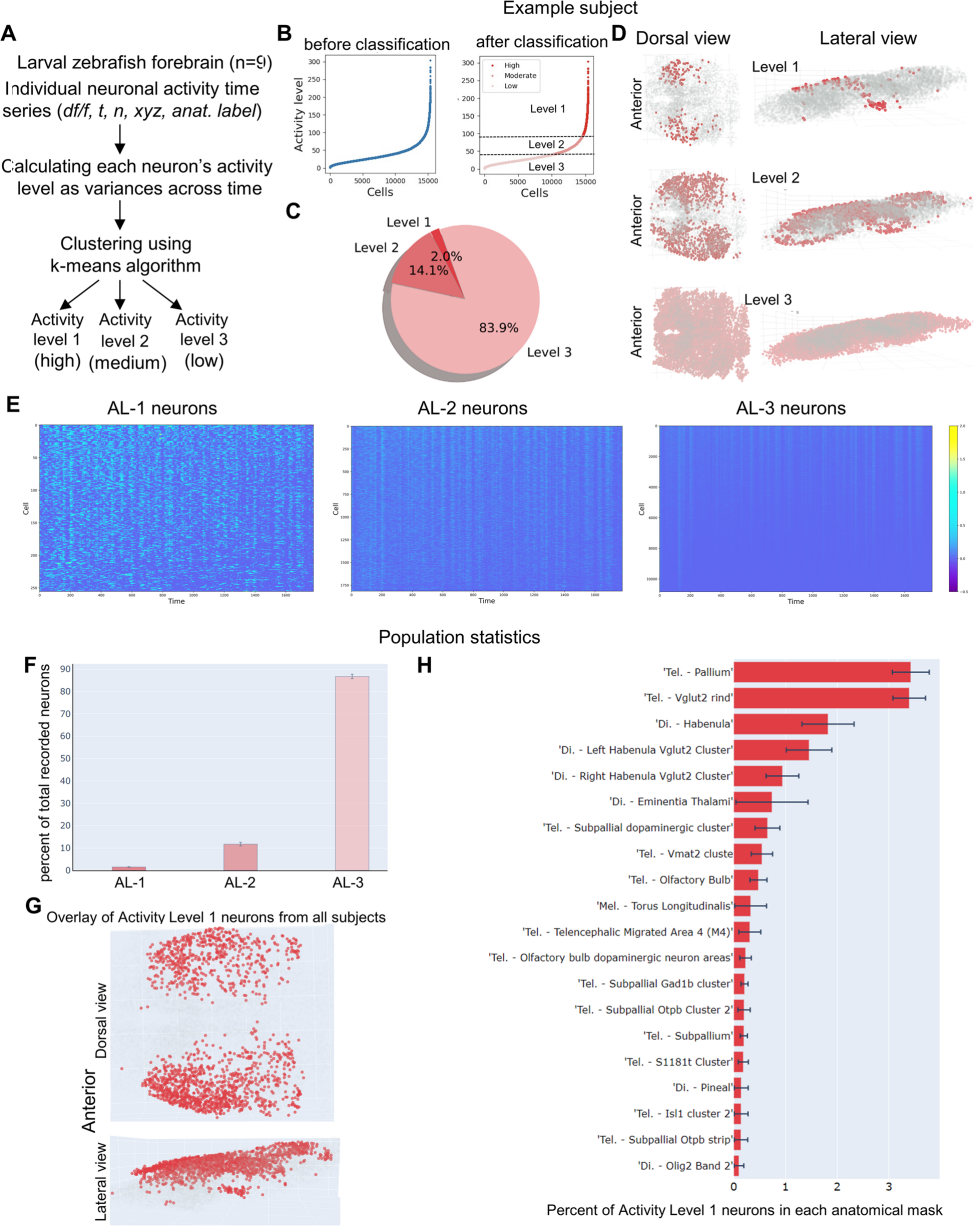
Visualization of neuronal activity landscape at cellular resolution in the larval zebrafish forebrain**. A** Overview of the classification of individual ROIs (neurons) based on their level of activity. The variance of df/f of each ROI was used as a measure of its activity. The k-means algorithm was used to classify each ROI into 3 levels. **B** Sorted ROIs (left) vs. clustered ROIs (right) based on their activity level for an example subject. **C** Pie chart showing the percentage of ROIs in three activity level categories for an example subject. Less than 2% of ROIs are highly active (level I) but more than 80% are largely inactive. **D** Dorsal and lateral views of the three activity categories of ROIs’ distributions in the example subject’s forebrain. **E** Raster plot of ROIs with different levels of activity in an example subject: (left) activity level 1 and (right) activity level 3. **F** Percent of total recorded neurons in each activity level category across 9 subjects. **G** Overlay view of highly active neurons (level 1) in all 9 subjects shows that they are located in the lateral part of the forebrain. **H** Anatomical distribution of activity level 1 neurons sorted based on the percentage of total recorded neurons in each anatomical mask. The number of replicates used is 9

As a first step toward data analysis, we visualized the neuronal activity landscape (Fig. 1A). Each neuron’s level of activity was calculated based on the variances of ΔF/F across time. The k-means clustering, which is a well-known unsupervised learning algorithm [49], was used to group neurons based on their activity levels. To distinguish neurons with the highest or lowest levels of activity, we set the number of clusters to 3. Hence, the activity levels of 1, 2, and 3 denoted neuronal groups with high, medium, and low activity. The activity level before and after classification was shown for an example subject (Fig. 1B). More than 80% of neurons were classified as Activity Level 3 (AL-3), whereas only ~2% of neurons belonged to Activity Level 1 (AL-1) (Fig. 1C). Visualization of their anatomical distribution showed that AL-1 neurons were mostly located in the lateral region of the forebrain, whereas AL-3 neurons were distributed in all brain areas (Fig. 1D). Raster plots of individual neuronal activity time series showed that our method was effective in separating neurons of high vs low activity (Fig. 1E). Similar observations were made across all subjects, as reflected by the population statistics (Fig. 1F) and the overlay view of AL-1 neurons from all subjects (Fig. 1G). Analysis of detailed anatomical distributions for AL-1 neurons showed that they are mostly located in the telencephalic pallium and diencephalic habenula (Fig. 1H). Together, these findings uncover highly active neurons that are located laterally in the larval zebrafish forebrain.

### Classification of neurons based on their degrees of functional connections in the larval zebrafish forebrain

The brain as a complex network involves intricate communications between individual neurons. An understanding of their patterns of communications will likely inform underlying network architectures. We therefore classified neurons based on their degrees of functional connections. Here, the degree or number of connections between a neuron and the rest of neurons in the dataset is used as a measure of functional connectivity, which can be approximated using various statistical measures. One common and effective measure for estimation of the connectivity matrix is the Pearson correlation coefficient value. We calculated the degree of connections for each neuron by applying optimal thresholding to the connectivity matrixes followed by binarization. The optimal thresholding value for each subject was determined using the principle of small world networks that follow a power law distribution [50, 51] (Additional file 4). Such power law distribution was not observable in randomly shuffled data (Additional file 5), indicating its biological relevance. Moreover, we showed that known connections between olfactory epithelial and olfactory bulb neurons were uncovered (Additional file 6), thereby validating our method of detecting functional connections.

**Fig. 2:**
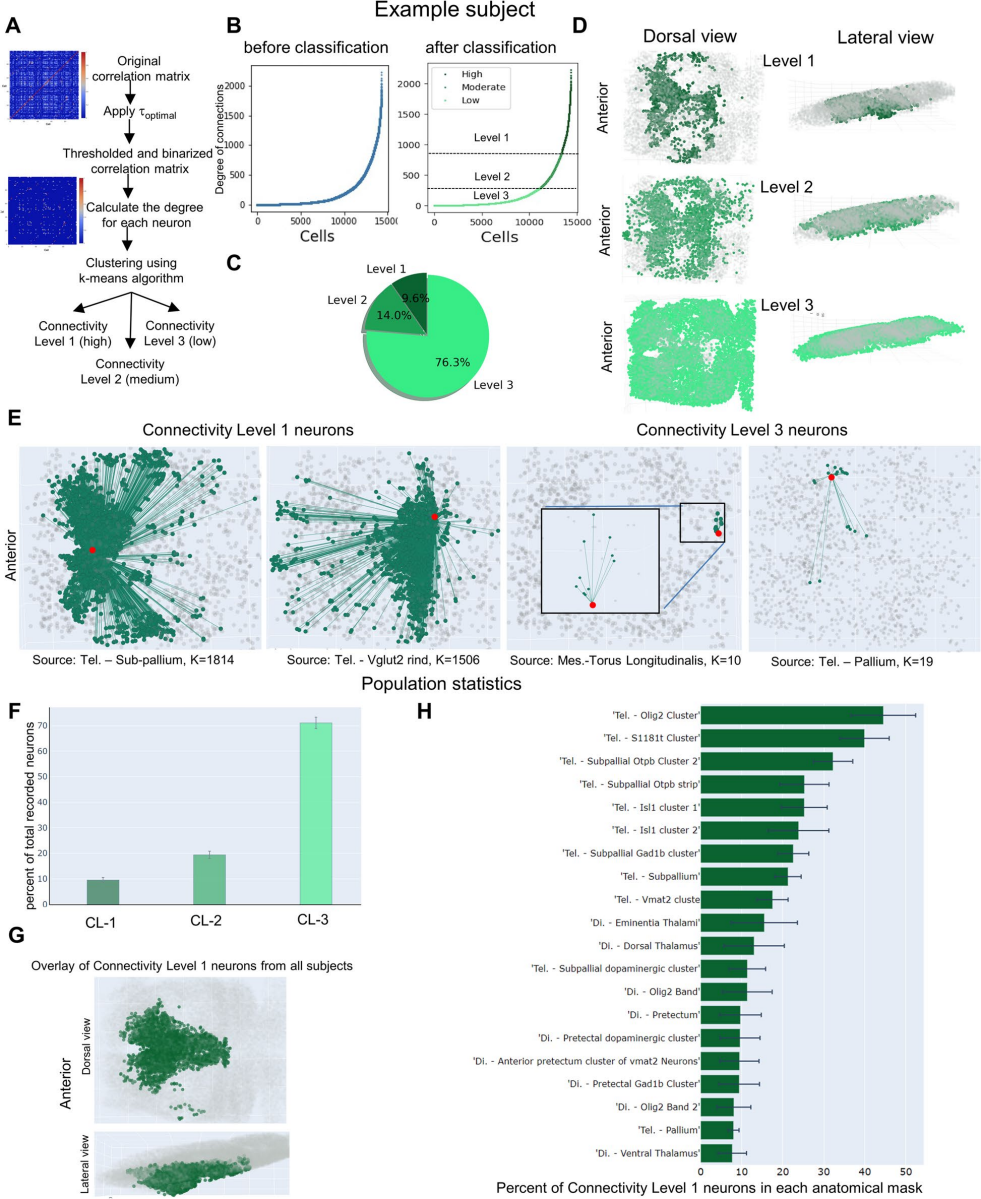
Classification of neurons based on their degree of functional connections. **A** Overview of the classification of individual ROIs (neurons) based on their level of functional connectivity (degree). The Pearson correlation coefficient was used to calculate the correlation matrix, which was then thresholded using the optimal threshold value. The k-means clustering algorithm was used to cluster ROIs based on their degree. **B** Sorted ROIs (left) vs. clustered ROIs (right) based on their functional connectivity level for an example subject. **C** Pie chart showing the percentage of ROIs in three connectivity level categories for an example subject. The ROIs with the highest level of functional connectivity is the smallest group (around than 8%). **D** Dorsal and lateral views of the three connectivity categories of ROIs’ distributions in the example subject's forebrain. **E** The connectivity of ROIs with the connectivity levels 1 and 3 in the example subject brain. **F** Percent of total recorded neurons in each functional connectivity level across 9 subjects. **G** Overlay view of highly functional connected ROIs (level 1) in all 9 subjects shows that they are located in the medial part of the forebrain. **H** Anatomical distribution of connectivity level 1 neurons sorted based on the percentage of total recorded neurons in each anatomical mask. The number of replicates used is 9

We next used the k-means algorithm to cluster neurons based on their degree of functional connections (Fig. 2A), with the number of clusters also set to 3. The connectivity level before and after k-means classification was shown for an example subject (Fig. 2B). ~8% of neurons belonged to connectivity level 1 (CL-1, the high connectivity group) whereas ~70% of neurons were classified as connectivity level 3 (CL-3, low connectivity) (Fig. 2C). Visualization of their anatomical distributions showed that the CL-1 neurons were mostly located in the medial area of the forebrain (Fig. 2C). Two example CL-1 neurons had 1814 and 1506 functional connections respectively, in contrast to two example CL-3 neurons with 10 and 19 connections, respectively, suggesting that our method is effective in separating neurons with high vs low connectivity (Fig. 2E). Consistent with the example subject, population statistics showed that the CL-1 neurons represent ~8% of total recorded forebrain neurons (Fig. 2F) and they are in the medial region of the forebrain (Fig. 2G). Analysis of detailed anatomical distributions for CL-1 neurons uncovered that Telencephalic Olig2 Cluster, Telencephalic S1181t Cluster, and Telencephalic subpallial Otpb strip are among the neuronal groups with high degrees of functional connections in the zebrafish forebrain (Fig. 2H). Together, these findings uncover neurons with high degrees of functional connectivity that are located medially/centrally in the larval zebrafish forebrain.

### Complementary domains of high neuronal activity and high functional connectivity exists in the larval zebrafish forebrain

**Fig. 3:**
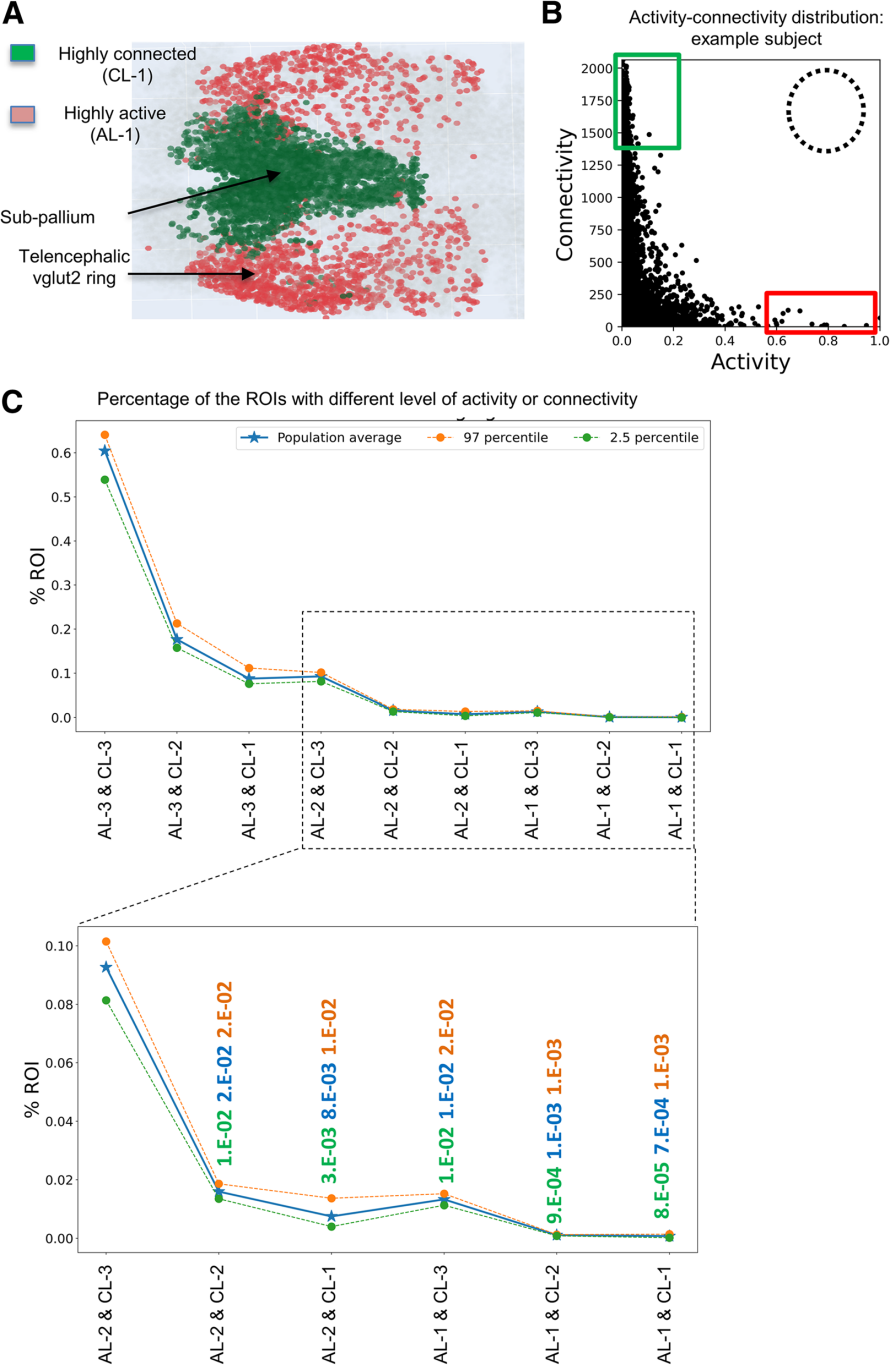
Highly active and highly connected neuronal populations occupy complementary domains in the larval zebrafish forebrain. **A** Overlay of highly active (red) and highly functional connected ROIs (individual neurons) in the larval zebrafish forebrain across 9 subjects. The highly active cells are in the lateral area whereas the cells with a high level of functional connectivity are located in the medial area. **B** Connectivity levels (*Y*-axis) of all neurons sorted based on their activity (*X*-axis) in an example subject. Red and blue boxes denote neurons of high activity and high functional connectivity, respectively. The dotted circle denotes where highly active and highly connected neurons are expected. **C** The population distribution curve of all neurons with different levels of activity and functional connectivity. Note that neurons that have high activity and high connectivity are non-existent. The number of replicates used is 9

It was intriguing to note that highly active neurons occupied regions that are complementary to those occupied by highly connected neurons in the larval zebrafish forebrain (Fig. 3A). Plotting the activity and functional connectivity values for all recorded neurons in one example subject showed that highly active neurons did not overlap with highly connected neurons (Fig. 3B). To visualize the distribution and assess statistical significance on a population scale, we used a bootstrapping method [52] to construct a graph showing the percentage of neurons with different levels of activity and connectivity within 95% confidence intervals (i.e., AL-1&CL-1, AL-1&CL-2, AL-1&CL-3, AL-2&CL-1, AL-2&CL-2, AL-2&CL-3, AL-3&CL-1, AL-3&CL-2, AL-3&CL-3). Specifically, 9 subjects were randomly sampled with replacement, and this was repeated at least 25 times. Neurons belonging to both AL-1 and CL-1 were undetected (Fig. 3C), confirming a mutually exclusive relationship between highly active and highly connected neurons in the larval zebrafish forebrain.

### Neuronal populations of high activity versus high functional connectivity are largely non-overlapping in the whole larval zebrafish brain

**Fig. 4:**
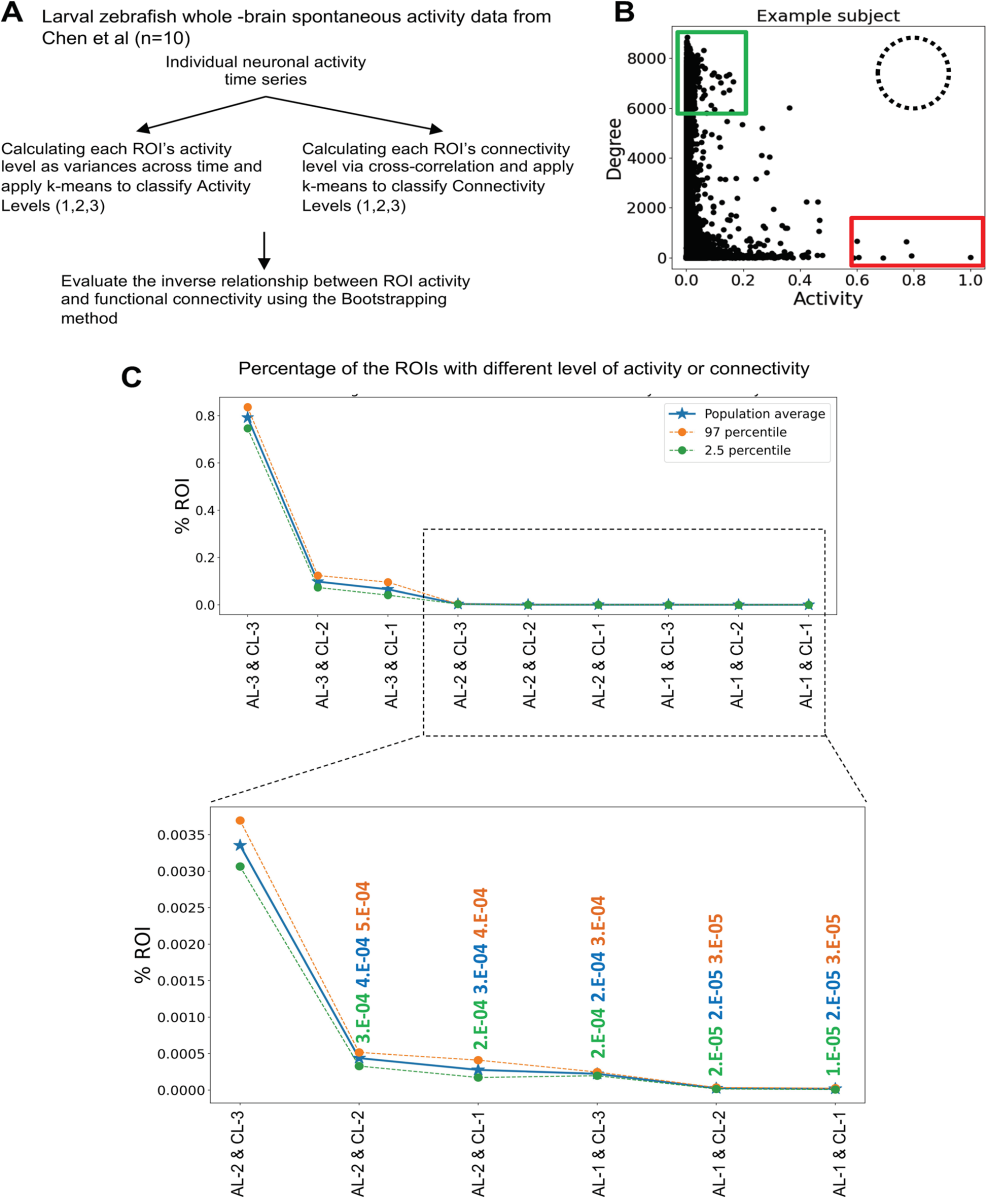
Non-overlapping distribution of highly active and highly connected neuronal populations in the whole larval zebrafish brain. **A** Schematic diagram showing the analysis pipeline applied to the whole brain spontaneous activity data from larval zebrafish (*n*=10). **B** Connectivity levels (*Y*-axis) of all neurons sorted based on their activity (*X*-axis) in an example subject. Red and blue boxes denote neurons of high activity and high functional connectivity, respectively. The dotted circle denotes where highly active and highly connected neurons are expected. **C** The population distribution curve of all neurons with different levels of activity and functional connectivity. Note that neurons that have high activity and high connectivity are non-existent. The number of replicates used is 10

We next wondered whether our observation in the zebrafish forebrain is applicable to the whole brain. The whole brain spontaneous activity data from Chen et al. [29] were analyzed. Individual neuronal activity time series were used to calculate each neuron’s activity and functional connections (Fig. 4A). By plotting their relationship, we found that, like the forebrain (Fig. 3B), neurons with both high activity and high connectivity were non-existent (Fig. 4B, represented by the empty black circle). Similarly, on a population scale, neurons that belonged to AL-1 (highest level of activity) and CL-1 (highest level of connectivity) approached zero (Fig. 4C). These results suggest that neurons that are both highly active and highly connected are undetectable in the whole larval zebrafish brain.

### K-means clustering of neurons into five clusters yields the same finding for both forebrain and whole brain data

To verify whether not detecting neurons that are both highly active and highly connected is due to clustering neurons into three clusters, we divided the data into five clusters and examined the relationship between activity and functional connectivity. The same trends were observed for both the forebrain and whole brain spontaneous activity data (Additional file 7), suggesting that our finding holds true regardless of the number of clusters generated by K-means analysis.

### Noise-added, shuffled, and simulated data show activity-connectivity relationships that are distinct from the real brain data

**Fig. 5:**
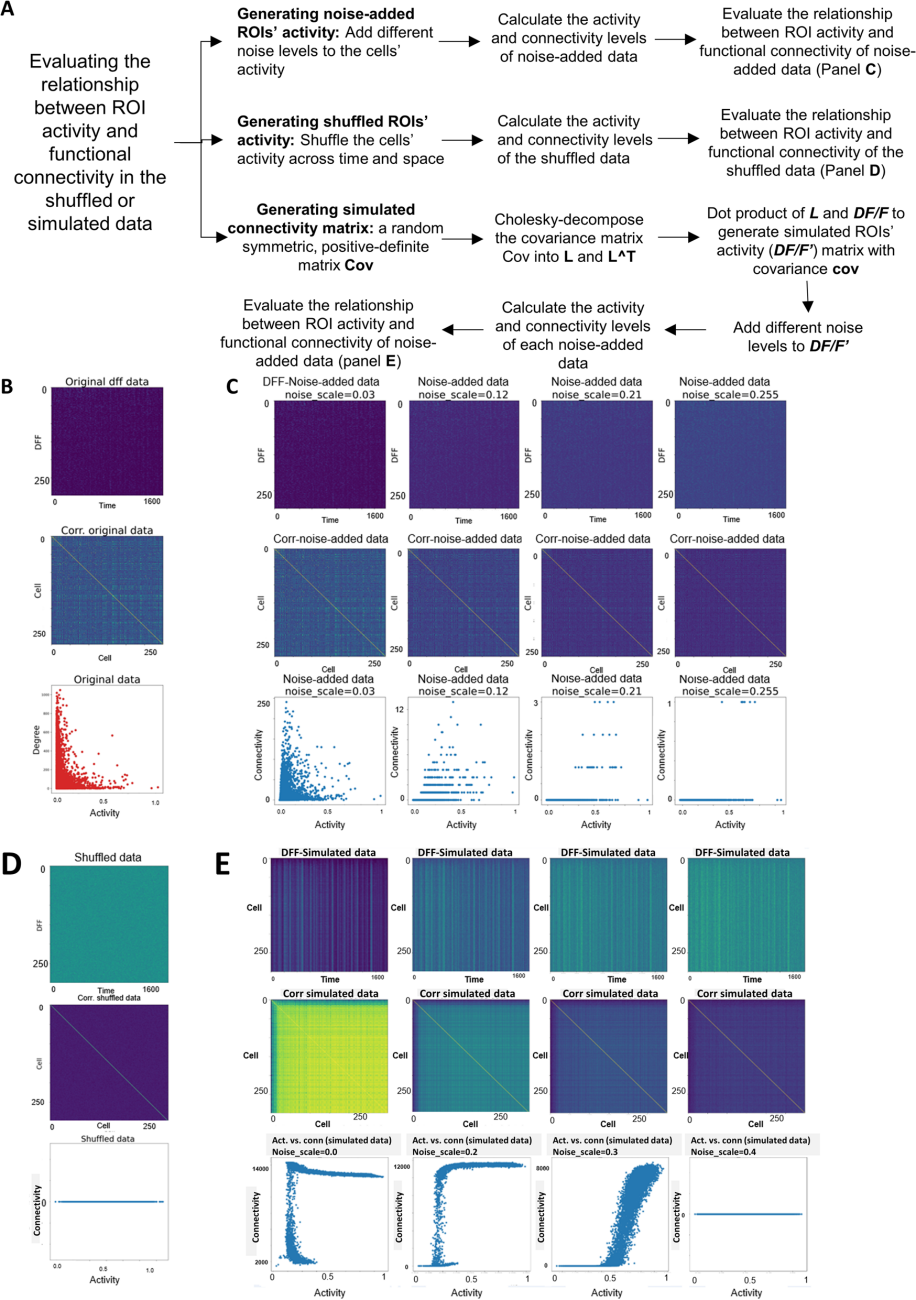
Shuffled, noise-added, and simulated data show activity-connectivity relationship that is distinct from the brain data. **A** Schematic showing the generation and analysis of noise-added, shuffled, or simulated data. **B**–**E** Different datasets with neuronal activity time series (top), cell-wise correlation matrix (middle), and the graphed functional connectivity and activity relationship (bottom). **B** Original data of an example subject. **C** Different levels of noise were added to the original data, resulting in the loss of the activity-connectivity relationship observed in the original brain data. Note that the *Y* axis range is different across the panels. **D** Neuronal activity time series of the original data were shuffled in both space and time. The activity-connectivity relationship observed in the original brain data was lost. **E** A simulated dataset shows the activity-connectivity relationship that is distinct to the brain data and is also sensitive to the levels of noise. The number of replicates used is 9

To determine whether the lack of ROIs that are both highly active and highly connected is unique to the zebrafish brain data, or whether it is a property of any sets of interconnected units, we applied our analytic pipeline to shuffled, noise-added, or simulated data. Three approaches were used to generate these datasets (Fig. 5A): First, we added different levels of noise to our brain data. Second, we shuffled neuronal activity time series across time and space. Finally, we generated a random covariance (i.e., connectivity) matrix and used the Cholesky method [53] to construct the corresponding activity matrix. We found that the activity-connectivity relationship changed drastically with increasing noises added to the real brain data (Fig. 5C). When the activity time series were shuffled in both time and space, functional connections were completely lost, resulting in connectivity of 0 regardless of neuronal activity (Fig. 5D). Finally, simulated data showed an intriguing relationship between activity and connectivity, such that when activity was above certain values, connectivity became very high, and such relationship was sensitive to noise addition (Fig. 5E). Taken together, these results suggest that the lack of both highly active and highly connected neurons is a real attribute to the zebrafish brain.

Regions of high neuronal activity versus high functional connectivity are largely non-overlapping in the resting state human brain.

**Fig. 6:**
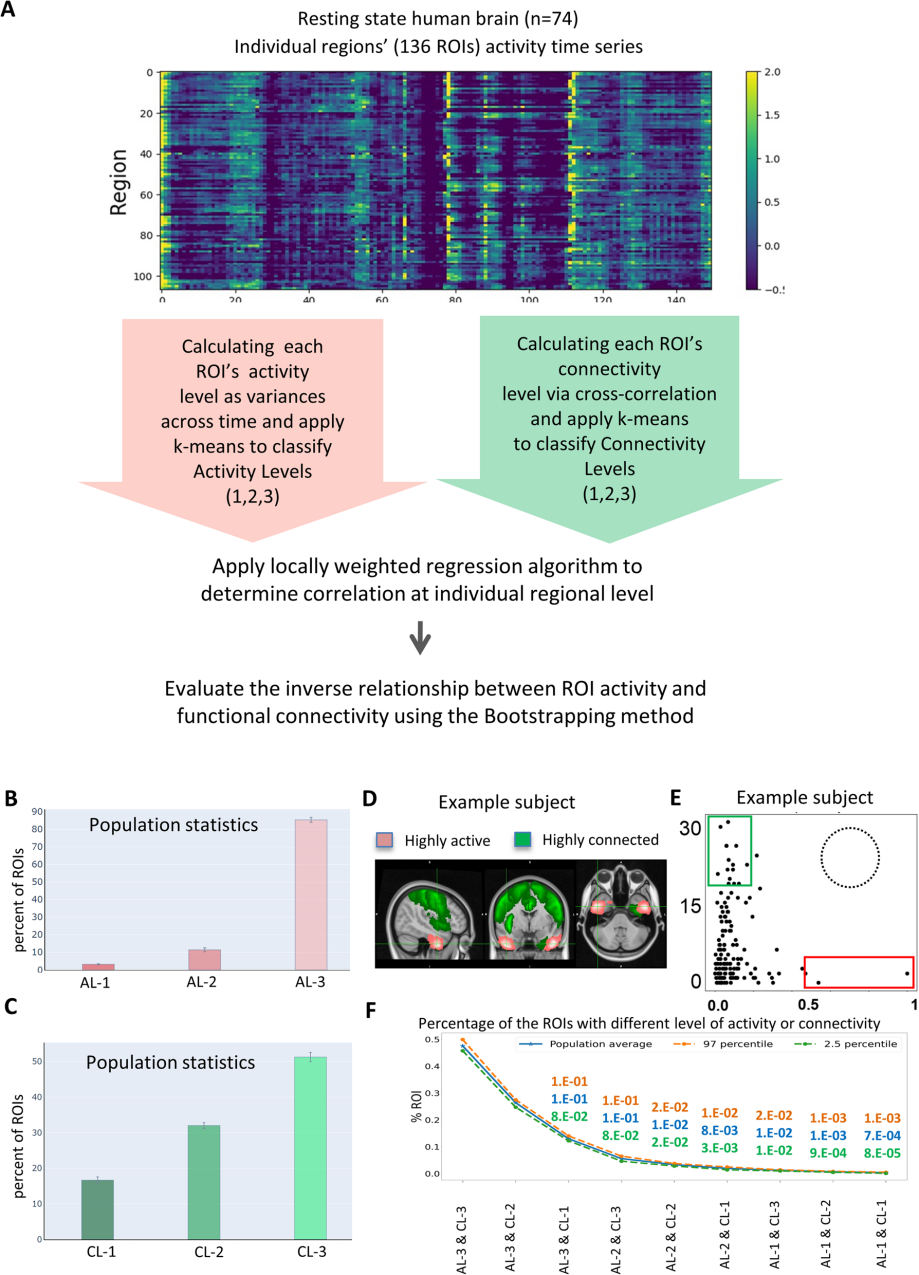
Largely non-overlapping distribution of highly active and highly connected regions in the resting state human brain. **A** Overview of the classification of individual ROIs (brain regions, *n*=105) based on their level of activity and functional connectivity (degree). Variations of the brain region activity across time was used as a measure of activity and the Pearson correlation of brain regions’ activity was used a measure of functional connectivity (degree). The k-means clustering algorithm was employed to cluster the brain regions into three levels based on each measure. **B** Percent of total ROIs in each activity level category. **C** Percent of total ROIs in each functional connectivity level category. **D** Highly active (red) and highly connected regions (green) across all subjects (*n*=74). **E** Connectivity levels (*Y*-axis) of all brain regions sorted based on their activity (*X*-axis) in an example subject. Red and blue boxes denote regions of high activity and high functional connectivity, respectively. The dotted circle denotes where highly active and highly connected brain regions are expected to locate. **F** The population distribution curve of brain regions with different levels of activity and functional connectivity. Note that brain regions that have high activity and high connectivity are non-existent. The number of replicates used is 74

To determine whether the observed relationship between activity and connectivity is an evolutionarily conserved phenomenon, we analyzed the resting state human brain functional magnetic resonance imaging (fMRI) data from Centre for Biomedical Research Excellence (COBRE) dataset [54] (Fig. 6A). The COBRE dataset includes the resting state fMRI data from 74 healthy individuals that were used in this study. The fMRI dataset for each subject includes volumes of blood-oxygenation-level-dependent (BOLD) signals of 5 minutes. The BOLD signal reflects changes in deoxyhemoglobin driven by localized changes in brain blood flow and blood oxygenation, which are coupled to underlying neuronal activity by a process termed neurovascular coupling [55]. In this study, we used the variances of BOLD signals over time as the ROI activity. This is analogous to our zebrafish calcium signal data analysis, where the variances of ΔF/F signals over time is measured as the ROI activity. While each ROI in the larval zebrafish data is a single neuron, each ROI in the human fMRI data is a brain region. Brodmann areas defined by the Talairach Daemon (TD) system [56] were assigned to all subjects. Data pre-processing workflow was shown in Additional file 8.

Using the strategy described above, we calculated the activity for each ROI, followed by applying the k-means algorithm to classify ROIs into three levels of activity (Fig. 6B and Additional file 9 A-B). Our analysis showed that the anterior division of inferior temporal gyrus displayed highest activity that was observed in more than 45% of the subjects (Additional file 9 C-D), whereas the hippocampus, pallidum, and putamen were less active in the resting state (Additional file 9 E and Table S1). The default mode network (DMN), which is primarily composed of the medial prefrontal cortex, posterior cingulate cortex/precuneus, and angular gyrus, is defined as having decreased activity during task performance compared to the resting state [57]. However, the extent of BOLD signal fluctuations in these regions in comparison to other brain regions is not clear. Based on our calculations of variances in BOLD signals, the DMN was not of highest activity at the resting state, with the medial prefrontal cortex ranked 16, posterior cingulate cortex ranked 112, and angular gyrus ranked 114 out of 132 ROIs analyzed. These observations suggest that the BOLD signal variances show heterogeneity in the DMN and are not the highest when compared to other brain regions.

We next used the Pearson correlation measure to derive a metric of functional connectivity between ROIs followed by k-means classification into three levels of connections (Fig. 6C and Additional file 10 A-B). Analogous to the analysis of functional connectivity in the zebrafish brain, we applied the thresholding value that provided the best fit to the power-law curve as the optimal threshold value for the connectivity matrix. Our analysis showed that the precentral gyrus, right postcentral gyrus, and anterior division of Cingulate Gyrus were among the regions with high connectivity in the human brain, whereas the Pallidum right frontal medial cortex, subcallosal cortex, and amygdala were much less connected in almost all subjects in the human resting state dataset (Additional file 10 C-E, Table S1). Consistent with previous reports that dorsal sensorimotor and attentional areas correlate more strongly with the global signal, while activity in the anterior temporal lobe correlates relatively less with the global signal [58, 59], we found that the highly connected regions, such as the precentral gyrus left/right, postcentral gyrus left/right, postcentral gyrus left, cingulate gyrus, anterior division, and lingual gyrus left, were strongly correlated with the global signal and were also strongly correlated with one another.

Like the observations made in the larval zebrafish brain, highly active regions and highly functionally connected regions appeared complementary and non-overlapping in the resting state human brain (Fig. 6D). Plotting the activity and functional connectivity values for all recorded brain regions in one example subject (Fig. 6E) and the percentage of ROIs in each activity and connectivity category across subjects (Fig. 6F) further reinforced this notion.

To determine whether the lack of ROIs that were both highly active and highly connected (as shown in Fig. 6E) was observable at threshold levels other than the optimal threshold used for deriving functional connections, we generated connectivity matrices of the example human subject using different threshold values and plotted the corresponding relationship between activity and functional connectivity (Additional file 11). The results showed that, at the values much lower than the optimal threshold value, many low activity ROIs had inflated connections and distributed toward the top left corner of the graph. On the other hand, at the values much higher than the optimal threshold value, connections between ROIs were lost. Therefore, our observed correlations are only found at the values approaching the optimal threshold. These observations suggest that our observed activity-connectivity relationship (as shown in Fig. 6E) likely reflects a real brain property that is not observed at arbitrarily low or high threshold values, which has likely captured noises or abolished real connections, respectively.

Additionally, to determine whether consistent results can be found without using a thresholding procedure in deriving functional connection measures, we calculated functional connectivity by using the sum of correlation values for each ROI (Additional file 12 A). We obtained similar results with (left) or without (right) a thresholding procedure in both zebrafish (Additional file 12 B) and human (Additional file 12 C) subjects.

Finally, to determine whether our observed activity-connectivity relationship (as shown in Fig. 6E and Additional file 13 A) is observable with any sets of interconnected units, we performed similar analysis as we did with the zebrafish data for the human data. We analyzed (1) human data with different levels of noises added (Additional file 13 B), (2) randomly shuffled data (with activity data shuffled in both space and time) (Additional file 13 C), and (3) simulated data containing similar numbers of ROIs to the human data (with different levels of noise added) (Additional file 13 D). Again, we observed activity-connectivity relationships that were distinct from the real brain data, suggesting that our findings represent a network property that is not observed in any interconnected datasets.

Together, like in the zebrafish brain, regions with high functional connectivity and regions of high activity appear mutually exclusive in the resting state human brain.

## Discussion

One major goal of neuroscience is to understand fundamental organizational principles of the brain. While functional imaging and analysis of brain networks in larval zebrafish is an emerging field, numerous studies of resting state human brain networks have examined brain activity or connectivity patterns, suggesting the prevalence of activity-based or connectivity-based organizations [37, 60].

Despite these advances, the relationship between activity and functional connectivity in the brain is not well understood. This is an interesting and important question both for understanding the brain architectural principles and for designing artificial neuronal networks. In this study, we have examined the relationship between activity and functional connectivity in both the larval zebrafish forebrain and whole brain where each ROI is an individual neuron and in the resting state human brain where each ROI is a brain region composed of millions of neurons. In larval zebrafish, activity is measured through quantifying variances of fluorescence signals emitted from the calcium indicators (ΔF/F) over time: more frequent events of ΔF/F changes are used to indicate higher neuronal activity. In the human brain, activity is measured through the BOLD signals. More frequent events of BOLD signal changes are used to reflect higher neuronal activity. Functional connectivity in both the larval zebrafish and human brains is measured using Pearson correlation; the resulting correlation matrices are further denoised with optimal threshold values, which are determined using the concept of “small-world” network with power law distribution. Through these analyses, we have uncovered a mutually exclusive relationship between ROIs of high activity and ROIs of high functional connectivity across all zebrafish and human subjects, such that ROIs of both high activity and high functional connectivity are not detected. This shared property between the zebrafish and the human brains is not observed with noise-added, shuffled, or simulated datasets, nor is it sensitive to the analytic methods (e.g., k-means clustering numbers or calculating functional connectivity with or without a threshold), suggesting that it reflects real brain property. This is remarkable, given the 450 million years of evolutionary distance and the drastic brain size differences (100K vs. 100 billion neurons) between the two species.

Although the relationship between activity and functional connectivity in the brain has been infrequently assessed, one previous study by Di et al. has examined the influence of the amplitude of low-frequency fluctuations (ALFF) on resting-state functional connectivity [61]. Di et al. evaluated the correlation between each ROI’s local ALFF, calculated in the low frequency band between 0.01 and 0.08 Hz, and its functional connectivity, and the correlations were thresholded at |r| > 0.364. The functional connectivity of several ROIs was found to correlate with its own regional ALFF. Our use of variances to infer ROI activity (covering a possibly broader frequency) is to a certain extent similar to ALFF, which measures the relative contribution of low frequency fluctuations (0.01–0.08 Hz) and is determined as the sum of amplitudes within a specific low frequency range [62]. Despite this, our analysis involves the ranking of all ROIs based on their activity and connectivity, which uncovered that the ROIs of high activity were not the ones of high connectivity. Di et al. did not take the same approach but instead reported the ROIs with correlated ALFF and functional connectivity. Therefore, our findings are different because of the different questions being asked, but they are not in any way inconsistent, as our findings do not exclude the possibility that the ROIs with medium/low activity and connectivity might have a positive correlation between the two measures.

In summary, our findings are unexpected in two ways. First, it is generally thought that a ROI with more connections likely display more activity fluctuations, hence higher activity based on our calculations of variances. Instead, we found the contrary. The highly active ROIs have few functional connections; thus, to achieve high activity, they must make frequent communications with their connected partners. Second, the brains of zebrafish and humans are different in many ways. In addition to the size and complexity, the resting state human brain is unlikely to be at the same level as the zebrafish brain, because humans are known to engage in many types of cognitive processes during “rest”. These processes include mind-wandering, self-reflection, and planning. What is going on in the resting state zebrafish brain is unknown but is unlikely to be the same as those in the human brain. Yet, we found a lack of ROIs that are both highly active and highly connected in both brains.

We propose two possible models to explain why such exclusive relationship between high activity and high functional connectivity is at work in both zebrafish and human brains. The first model pertains to a physical constraint. Given that structural and functional connectivity show considerable correlation [18], it is possible that neurons with high levels of connections are physically incapable of achieving high activity. The second model is based on a metabolic constraint. ROIs with high activity are at a high metabolic cost [63], thereby accumulating more oxidative damage and prone to degeneration. To best preserve network integrity, it would therefore be desirable to delegate the tasks that require high activity to the ROIs with low degrees of functional connections, while maintaining ROIs with high connections at low activity. Future experiments are necessary to test these models. With the accessibility of the zebrafish brain to molecular cellular and systems level dissections, such validations may be feasible in zebrafish.

## Conclusions

By analyzing brain-wide calcium imaging and fMRI data, we found a mutually exclusive relationship between high activity (signal variance over time) and high functional connectivity of neurons in zebrafish and human brains. These findings reveal a previously unknown and evolutionarily conserved brain organizational principle that have implications for understanding disease states and designing artificial neuronal networks.

## Methods

### Zebrafish strain maintenance and larval sample preparation

The transgenic line *Tg[HuC-H2B-GCaMP6s]* with *nacre* or *casper* background was used for breeding. Embryos were kept in blue egg water (2.4 g of CaSO4, 4g of instant ocean salts, and 600μl of 1% methylene blue in 20 l of milliQ water) and incubated at 28°C. On 6 days post-fertilization (dpf), healthy larvae with high GCaMP6s expression were selected for imaging. Fish samples are held in custom-designed polydimethylsiloxane (PDMS) sample holders with each holder carrying up to 5 larvae. Each larva was half-embedded in a slot on the sample holder, paralyzed with 1mg/mL mivacurium chloride, and covered with 2% agarose gel/E3 medium solution. After loading the whole group of larvae to be imaged, the sample holders were immersed in E3 medium for 1 h to wash off traces of mivacurium chloride. All animal experiments were approved by the Institutional Animal Care and Use Committee (IACUC) at the University of California, San Francisco, USA.

### In vivo calcium imaging using iSPIM

An inverted SPIM (iSPIM) that is similar to a previously reported design [41] was used for imaging. The microscope framework was adapted from a di-SPIM (Applied Scientific Imaging, Inc.) [64]. The excitation laser (Coherent OBIS LS 488 nm) was fiber-coupled into the microscope, collimated, then focused by a 0.3 NA water-dipping objective (Nikon). A virtual light-sheet was created by deflecting the scanning mirror, illuminating a layer of specimen with ~7-μm thickness. Fluorescence from the illuminated layer was collected in an orthogonal direction by a 20x 1.0 NA water-dipping objective (Olympus XLUMPLFLN-W), and the final image is captured by a scientific CMOS (sCMOS) camera (Hamamatsu Orca Flash 4.0, C11440). Due to the strong scattering of the blue-green light in deep tissues, we confined the illumination within dorsal forebrain of the larvae. The volume of interest was approximately 300 μm x 250 μm x 100μm in *x*,*y*,*z* directions, respectively. This volume covered the entire dorsal telencephalon and habenula regions. To resolve the dynamics of GCaMP6s, image stacks were acquired at 2Hz, which allowed us to resolve frequency components up to 1Hz based on Nyquist sampling theorem. Each 100-μm stack consisted of 26 slices with 4 μm between two adjacent slices. The resting state of the selected volume in each larva was imaged for 15 min; in addition to this time lapse recording, a Z-stack with 1-μm step (101 slices) was acquired across the same volume as a reference. The raw data were submitted to the data preprocessing pipeline for cleaning and feature extraction.

### Pre-processing of calcium imaging data

#### Drift correction and ROI (neuron) extraction

Raw images were organized as hyper stacks in the order of x-y-z-t. Each hyper stack was split into 26T-stacks at different *z* positions and drift corrected with the StackReg plugin [65]. For each drift-corrected T-stack, neuronal nuclei were segmented using the Laplacian of gaussian blob detection algorithm blob_log in the Python library scikit image [66]. Since neuronal nuclei (~4μm in diameter) can be imaged in two or more adjacent planes, a redundancy detection algorithm was developed to find lateral duplications in cell extraction: if a nucleus is detected at the same (*x*; *y*) position in the kth and the (k + 1)th planes, this detection would be considered as redundant and the Z-position was considered as an average between zk and zk+1. In each extracted neuronal nuclei, its raw fluorescence signals were calculated through the entire T-stack, and the relative signal intensity of calcium transients, ΔF/F, was calculated using the method as previously described [47]. Two additional cleaning steps were applied to remove potential artifacts that were falsely recognized as neuronal nuclei by the blob detection algorithm: first, since GCaMP6s has background signals in the absence of action potentials, blobs of real neurons should have a high fluorescence baseline, and blobs with very low baseline (comparable to dark areas in the image) are excluded; second, since in reality, the value of ΔF/F should fall within a reasonable range, blobs with extraordinarily high ΔF/F values (exceeding a threshold during recording) were excluded.

Since activity levels vary among neurons, over the 15-min imaging session, some neurons may exhibit high calcium signal peaks while others remain “silent”. Since the latter are not likely to contribute to downstream analyses, it would be beneficial to exclude them from the very beginning. This requires us to (1) find a reliable method to identify peaks from background in a noisy timeseries; (2) find a measure for the activity level of each neuron, i.e., whether and how much does it activate during the imaging session; and (3) set a well-defined criterion to accept or reject a neuron based on its two characteristics above. The method we used to identify peaks and baselines from each ΔF/F time trace was based on the Bayesian inference of two-dimensional distribution of adjacent ΔF/F values [33]. For each neuron *i*, its baseline of ΔF/F, μ_i_, was calculated by averaging all the (ΔF/F)_I_ time points that are identified as background,

$$\left|{a}_i\right.\sum \limits_k\left({S}_{i,k}-{\mu}_i\right)\times \Delta t,$$

where S_i,k_ refers to the *k*th time point of (ΔF/F)_i_.

#### Image registration

Since individuals differ in morphology, placement, and brightness, it is difficult to directly compare their images even though the latter are acquired under the same condition. In order to compare imaging results from different individuals, the images should be anatomically mapped to a common template image, i.e., a “reference”. A whole-brain template, Z-brain, has been provided by Randlett et al. [46] as a standard reference brain atlas for anatomical and functional studies of larval zebrafish brain. Although the fish we experimented on were at the same stage as that in the Z-brain template, the iSPIM stacks acquired in their dorsal forebrain could not be directly registered to the reference due to (1) a significant mismatch between two imaging directions and (2) a significant difference between the volumes of interests. Since the dorsal forebrain region that we imaged only accounts for a sub-volume of the entire brain, a direct registration of the former to the latter is prone to error. To solve this problem, we created an intermediate reference brain from the Z-brain template by resampling the dorsal-forebrain region in the direction and pixel sizes that are comparable to those of the iSPIM stacks. For each fish, its densely sampled Z-stack was registered to the intermediate reference brain using computational morphometry toolkit (CMTK) (http://nitrc.org/projects/cmtk). After registering the iSPIM stacks into the intermediate template, the coordinates of the extracted neurons in original T-stacks need to be reformatted accordingly into the registered frame. This step was carried out using the registration output and the function stream x form in CMTK. By comparing the reformatted coordinates with the 294 anatomical masks in the Z-brain template, we were able to identify the anatomical label of each neuron and the brain regions covered by our imaging volume.

### Preprocessing of the resting state human brain fMRI data

We used healthy control subjects (*n*=74) from the COBRE^16^ resting-state fMRI datasets available at http://fcon_1000.projects.nitrc.org/indi/retro/cobre.html. The fMRI dataset for each subject includes blood-oxygenation level-dependent (BOLD) volumes of 5 min (TR = 2 s, TE = 29 ms, FA = 75°, 32 slices, voxel size = 3x3x4 mm^3^, matrix size = 64x64, FOV = 255 x255 mm^2^). The pre-processing steps included realignment, co-registering, and normalization. We used established preprocessing and analysis pipelines [67] and CONN software package (https://www.nitrc.org/projects/conn) to estimate and remove noise components such as those from cerebrospinal fluid, white matter signals, and subject motion. The temporal band-pass filter (between 0.008 and 0.09 Hz) was applied to remove frequencies that were not of interest in the raw data. In addition to the default denoising strategy, data-driven Independent Component Analyses (ICA) denoising [68] was utilized to detect and remove potential noise-related temporal components.

#### Re-alignment

In brief, the first-level covariate containing the 6 rigid-body parameters was created based on the MRI data to estimate the subject motion. For each subject, this variable was used to perform regression on the fMRI data to correct for motion-related effects.

To reduce the physiological noise source, a Component-Based Noise Correction Method (CompCor) was used [69].

#### Co-registering

The functional volumes are co-registered with the ROIs and structural volumes. All the Brodmann areas (ROIs) defined through the Talairach Daemon (TD) system [56] were assigned to all subjects using segmentation of structural image; gray matter, white matter, and cerebrospinal fluid (CSF) masks were generated. Anatomical volumes were co-registered to the functional and ROI volumes for each subject, and the volumes were transformed to the MNI-space.

#### Calculation and normalization of fMRI measures

Following re-alignment and co-registering, the Principal Component Analysis (PCA) algorithm was used to extract BOLD signal components for each ROI. The fMRI measures were calculated using MATLAB-based software packages, SPM12 (http://www.fil.ion.ucl.ac.uk/spm/). All of the computed measures are normalized to an N (0,1) Gaussian distribution for each subject.

### Data analysis

#### Analysis and classification of ROI activity levels

For a given ROI (i.e., individual neurons in larval zebrafish or individual brain regions in the human fMRI data), signal *s*_*i*_ represents calcium indicator fluorescence changes (ΔF/F) in the zebrafish data or BOLD signals in the human fMRI data over time, the activity value *ac*_*i*_ was calculated as follows:

$${ac}_i= mean\ \left({\left({s}_i- mean\left({s}_i\right)\right)}^2\right)$$

After obtaining each ROI activity level in the given time series, we used the K-means algorithm [49] to classify them into activity levels 1–3 (or 1-5). The k-means clustering algorithm minimizes the within-cluster squared Euclidean distances. Here, a one-dimensional activity population was partitioned into 3 sets (levels). The within-class cells in each level have similar activity.

##### Analysis and classification of ROI functional connectivity levels

We used Pearson correlation to measure functional connectivity between ROIs. Since Pearson correlation assigns a value to all ROI pairs, it is necessary to apply thresholding to eliminate potentially spurious connections. There is no standard method to calculate the optimal threshold value *τ*_*optimal*_, and different values of *τ* are used to create the adjacency matrices. Arbitrarily chosen thresholding values are often applied to raw matrixes [6]. As different cutoff values can directly influence network properties and bias analysis results, we developed algorithms to calculate the optimal thresholding values and generate the connectivity matrix. This matrix was then used to calculate the functional connections (i.e., degrees) of each ROI, followed by K-means classification into three connectivity levels. The steps of calculating functional connections for each ROI are as follows:

1. Let *ρ* = *ρ*_*ij*_ be the correlation matrix, where *ρ*_*ij*_ is the Pearson correlation of ROIs *i* and *j* and can be calculated as follows:

$${\rho}_{ij}=\frac{\mathit{\operatorname{cov}}\left(i,j\right)}{\delta_i{\delta}_j},$$

2) Setting the threshold values:

$${\displaystyle \begin{array}{c}\tau =\left({\tau}_k\right)\\ {}{\tau}_k\in \left(0,1\right)\end{array}}$$

3) Thresholding the connectivity matrix using *τ*_*k*_ ∈ *τ* or each *ρ*_*ij*_
  $${\rho}_{\begin{array}{c}{\tau}_k\\ {} ij\end{array}}=\left\{\begin{array}{c}{\rho}_{ij}\ if\ {\rho}_{ij}>{\tau}_k\\ {}0\ otherwise,\end{array}\right.$$

where $${\rho}_{\tau_k}=\left({\rho}_{\begin{array}{c}{\tau}_k\\ {} ij\end{array}}\right)$$ is the thresholded correlation matrix.

4) Binarizing $${\rho}_{\tau_k}$$
  $${\rho}_{\begin{array}{c}{\tau}_{k,b}\\ {} ij\end{array}}=\left\{\begin{array}{c}1\kern0.5em if\ {\rho}_{ij}>{\tau}_k\\ {}0\ otherwise,\end{array}\right.$$
5) Calculating the degrees for each *ROI*_*i*_, *degree*_*i*_ = ∑_*j*_*l*_*i*, *j*_ , where *l*_*i*, *j*_ is the link between *roi*_*i*_ and *roi*_*j*_.
6) Calculating the degree distribution of the network. The fraction of ROIs with the degree *k* is defined as follows:
  $${P}^{\tau_k}(k)=\frac{n_k}{n},$$

where *n*_*k*_ is the number of the ROIs that have degree *k*.

7) Calculating the fitting value of the degree distribution with the power-law distribution. *r*^2^ (coefficient of determination) was used to evaluate the closeness of data at each threshold value to the power-law curve.
8) Derive the optimal threshold value: At the optimal threshold value, the *r*^2^ is highest, which indicates the best fit of the data to the power-law curve.
  $${\tau}_{optimal}^{pw}\approx \mathit{\arg}\ {\mathit{\max}}_r\ \left({r}^2\right)$$

The detailed steps of calculating the optimal threshold value of the connectivity matrix were provided in the Additional file 4. The average ${\tau}_{optimal}^{pw}$value of all zebrafish subjects in our data ranged from 0.4 to 0.6. We applied this algorithm to the human fMRI data and obtained 0.7 for ${\tau}_{optimal}^{pw}$. Using these values, we binarized our data: correlations with a value less than ${\tau}_{optimal}^{pw}$ were set to zero, whereas those with a value greater than ${\tau}_{optimal}^{pw}$ were set to one.

To further test whether the observed power-law structure of the functional brain is relevant, we shuffled the data (Additional file 5) to generate random networks with the same numbers of nodes and edges as the original networks and applied similar thresholding and analysis of degree distributions. The random network did not follow a power law structure at any thresholding values tested. Together, these analyses enable us to establish optimal thresholding values that uncover biologically relevant networks. Using such matrixes, we were able to detect known connections between the olfactory epithelia and the olfactory bulb (Additional file 6).

After obtaining each ROI’s numbers of functional connections in the given time series, we used the K-means algorithm as described above to classify them into connectivity levels 1–3 (or 1-5).

##### Analysis of the relationship between activity and functional connectivity

We plotted the activity and connectivity for each ROI in each individual. The input data are zebrafish calcium imaging data are composed of >12k individual neuronal activity and connectivity per subject (*n*=9 subjects); human resting-state fMRI data are composed of 136 brain regions’ activity and connectivity per subject (*n*=74 subjects).

To analyze the population frequency of each class of ROIs (i.e., AL-1&CL-1, AL-1&CL-2, AL-1&CL-3, AL-2&CL-1, AL-2&CL-2, AL-2&CL-3, AL-3&CL-1, AL-3&CL-2, AL-3&CL-3) and its statistical significance, we used the following bootstrapping method:

1. Select the number of the bootstrapping iteration (here, 25 iterations were used).
2. Repeat the steps “3” to “7” 25 times for the input data.
3. Select a sample set with replacement from the set of all subjects.
4. Calculate activity (variances) and connectivity (degrees) data for each ROI from all sampled subjects.
5. Calculate activity (variances) and connectivity (degrees) data for each ROI from all sampled subjects.
6. Calculate the average ROI population size for each activity and connectivity level.
7. Calculate the mean, lower (2.5 percentile), and upper (97.5 percentile) point-wise confidence bands for the populations that are calculated in the step “6”.

### Generation of shuffled data, noise-added data, and simulated data

#### Generation of shuffled data

For the zebrafish data, a given ∆*F*/*F* signal matrix *D*_*C* × *T*_ with *C* rows (*C* ROIs) and *T* columns (*T* time stamps) was used to generate a new matrix $\hat{D_{C\times T}}$ by random shuffling across rows and columns with the NumPy library of Python programming language [70]. For the human data, a given BOLD signal matrix was used and shuffled the same as for the zebrafish data.

#### Generation of noise-added data

To generate noise-added data, we first generated a noise matrix ${\mathrm{N}}_{\mathrm{C}\times \mathrm{T}}^{\mathrm{s}}$with normal (Gaussian) distribution and standard deviation (noise scale) *s* to produce different levels of noise, using the NumPy library. This noise matrix was then added to a given ∆F/F matrix D_C × T_ (C cells and T time stamps) (in the case of zebrafish data) or a given BOLD signal matrix (in the case of human data). The noise-added data $\overline{D_{C\times T}}$ were as follows:

$$\overline{D_{C\times T}}={D}_{C\times T}+{N}_{C\times T}^s$$

#### Interconnected network simulation and approximation of the correspond ROI activity using the Cholesky decomposition technique

This method consists of two steps: First, we generated a random covariance (connectivity) matrix using the Python scikit library [71]. In order to apply the Cholesky method to construct a ROI activity matrix from the connectivity matrix, the starting connectivity matrix must be symmetric positive-definite in linear algebra [53]. Second, we created a ROI activity matrix that matched the above connectivity matrix, using the Cholesky method (53). The connectivity matrix *C*_*Cell* × *Cell*_ was decomposed into two parts: L and L^T^. We then generated a random matrix (cell x time). For any given random matrix *X*_*Cell* × *Time*_, the simulated ROI activity time series matrix *S*_*Cell* × *Time*_ was as follows:

$${S}_{Cell\times Time}={L}_{Cell\times Cell}{X}_{Cell\times Time},$$

#### Data visualization

Different python libraries were used to visualize the results. The plotly libraries (https://plotly.com/python/) were used to visualize the cells’ anatomical and spatial distributions in the calcium imaging data. We used the FSL (https://fsl.fmrib.ox.ac.uk/fsl) to visualize the brain regions in the fMRI data.

#### Statistical analysis

Sample sizes and statistics are reported in the figure legends and text for each measurement. To determine the relationship between activity and functional connectivity at individual ROI levels (Figs. 3c and 4e), we used bootstrap tests (with 7 iterations) to test whether the negative correlation between neuronal activity and functional connectivity is consistent across subjects. For each subject, the activity (variances) and connectivity (degrees) for each ROI were calculated. Individual neurons in the larval zebrafish calcium imaging data and individual brain regions in the human fMRI data were considered as ROIs. The ROIs were sorted based on their activity for each subject (*X*-axis). The connectivity of sorted ROIs for each subject (*Y*-axis) was then graphed. The locally weighted regression algorithm [72] was applied for each subject’s data to approximate the polynomial curve. Finally, the bootstrapping method [52] was used to evaluate the inverse relationship between neuronal activity and functional connectivity. Here, random sampling with replacement was applied for selecting the curves fitting of individual subjects to evaluate the model.

## Supplementary Information

**Additional file 1.** Light-sheet imaging and image processing generate large-scale single neuron activity data.

**Additional file 2.** Brain registration enables comparison of single-neuron activity across different individuals.

**Additional file 3.** Evaluation of the accuracy and representation of anatomical label assignment.

**Additional file 4.** Identification of optimal threshold values to uncover significant functional connections in larval zebrafish calcium imaging data.

**Additional file 5.** Analysis of the shuffled larval zebrafish calcium imaging data does not show power law distribution at any thresholding values.

**Additional file 6.** Validation of detected functional connections in the larval zebrafish forebrain.

**Additional file 7.** Highly active and highly connected neuronal populations are largely non-overlapping when analyzed using 5 clusters.

**Additional file 8.** A flowchart showing the human brain data preprocessing and analysis pipeline.

**Additional file 9.** Characterization of activity in the human brain.

**Additional file 10.** Characterization of the connectivity in the human brain.

**Additional file 11.** Activity-connectivity relationship of example human data at different threshold levels for the connectivity matrix.

**Additional file 12.** The mutually exclusive relationship between high activity and high functional connectivity is observed when deriving functional connectivity without using a threshold.

**Additional file 13.** Relationship between activity and connectivity in the shuffled or noise-added human data or simulated data.

**Additional file 14.** Video S1.

**Additional file 15.** Table S1.

## Data Availability

All data generated or analyzed during this study are included in this published article and supplementary information files, and additionally available at 10.6084/m9.figshare.19401128.v1

## Abbreviations

AL: Activity level
BOLD: Blood-oxygen-level-dependent
CSF: Cerebrospinal fluid
CompCor: Component-Based Noise Correction Method
CL: Connectivity level
DMNs: Default mode networks
Dpf: Days post-fertilization
EEG: Electroencephalography
EM: Electron microscopy
fMRI: Functional magnetic resonance imaging
GCaMP: Calcium indicator formed by GFP
GCaMP6s: Version 6 “slow” form the GCaMP genetically encoded calcium indicator
GFP: Green fluorescent protein
ISPIM: Inverted SPIM
MEG: Measures muscle response
PCA: Principal Component Analysis
PDMS: Polydimethylsiloxane
RSNs: Resting state networks
SPIM: Selective-plane illumination microscopy
TD: Talairach Daemon
WT: Wild type

## References

[1] White JG, Southgate E, Thomson JN, Brenner S (1986). The structure of the nervous system of the nematode Caenorhabditis elegans. Philos Trans R Soc Lond A.

[2] Meinertzhagen IA (2016). Connectome studies on Drosophila: a short perspective on a tiny brain. J Neurogenet.

[3] Vanwalleghem GC, Ahrens MB, Scott EK (2018). Integrative whole-brain neuroscience in larval zebrafish. Curr Opin Neurobiol.

[4] Chuang KH, Nasrallah FA (2017). Functional networks and network perturbations in rodents. Neuroimage.

[5] Sporns O, Betzel RF (2016). Modular brain networks. Annu Rev Psychol.

[6] Rubinov M, Sporns O (2010). Complex network measures of brain connectivity: uses and interpretations. Neuroimage.

[7] Bullmore E, Sporns O (2009). Complex brain networks: graph theoretical analysis of structural and functional systems. Nat Rev Neurosci.

[8] Lovett-Barron M, Andalman AS, Allen WE, Vesuna S, Kauvar I, Burns VM, Deisseroth K (2017). Ancestral circuits for the coordinated modulation of brain state. Cell.

[9] Oikonomou G, Altermatt M, Zhang RW, Coughlin GM, Montz C, Gradinaru V, Prober DA (2019). The serotonergic raphe promote sleep in zebrafish and mice. Neuron.

[10] Oh SW, Harris JA, Ng L, Winslow B, Cain N, Mihalas S, Wang Q, Lau C, Kuan L, Henry AM (2014). A mesoscale connectome of the mouse brain. Nature.

[11] Zingg B, Hintiryan H, Gou L, Song MY, Bay M, Bienkowski MS, Foster NN, Yamashita S, Bowman I, Toga AW (2014). Neural networks of the mouse neocortex. Cell.

[12] Hagmann P, Cammoun L, Gigandet X, Meuli R, Honey CJ, Wedeen VJ, Sporns O (2008). Mapping the structural core of human cerebral cortex. PLoS Biol.

[13] Hildebrand DGC, Cicconet M, Torres RM, Choi W, Quan TM, Moon J, Wetzel AW, Scott CA, Graham BJ (2017). al e: Whole-brain serial-section electron microscopy in larval zebrafish. Nature.

[14] Kasthuri N, Hayworth KJ, Berger DR, Schalek RL, Conchello JA, Knowles-Barley S, Lee D, Vázquez-Reina A, Kaynig V (2015). al e: Saturated reconstruction of a volume of neocortex. Cell.

[15] Wanner AA, Genoud C, Masudi T, Siksou L, Friedrich RW (2016). Dense EM-based reconstruction of the interglomerular projectome in the zebrafish olfactory bulb. Nat Neurosci.

[16] Briggman KL, Helmstaedter M, Denk W (2011). Wiring specificity in the direction-selectivity circuit of the retina. Nature.

[17] Hebb DO (1949). The organization of behavior.

[18] Honey CJ, Sporns O, Cammoun L, Gigandet X, Thiran JP, Meuli R, Hagmann P (2009). Predicting human resting-state functional connectivity from structural connectivity. Proc Natl Acad Sci U S A.

[19] Bentley B, Branicky R, Barnes CL, Chew YL, Yemini E, Bullmore ET, Vértes PE, Schafer WR (2016). The multilayer connectome of Caenorhabditis elegans. PLoS Comput Biol.

[20] Gaudet I, Hüsser A, Vannasing P, Gallagher A (2020). Functional brain connectivity of language functions in children revealed by EEG and MEG: a systematic review. Front Hum Neurosci.

[21] Chen TW, Wardill TJ, Sun Y, Pulver SR, Renninger SL, Baohan A, Schreiter ER, Kerr RA, Orger MB, Jayaraman V (2013). Ultrasensitive fluorescent proteins for imaging neuronal activity. Nature.

[22] Huisken J, Swoger J, Del Bene F, Wittbrodt J, Stelzer EH (2004). Optical sectioning deep inside live embryos by selective plane illumination microscopy. Science.

[23] Keller PJ, Schmidt AD, Wittbrodt J, Stelzer EH (2008). Reconstruction of zebrafish early embryonic development by scanned light sheet microscopy. Science.

[24] Chen BC, Legant WR, Wang K, Shao L, Milkie DE, Davidson MW, Janetopoulos C, Wu XS, Hammer JA, Liu Z (2014). Lattice light-sheet microscopy: imaging molecules to embryos at high spatiotemporal resolution. Science.

[25] Ahrens MB, Li JM, Orger MB, Robson DN, Schier AF, Engert F, Portugues R (2012). Brain-wide neuronal dynamics during motor adaptation in zebrafish. Nature.

[26] Ahrens MB, Orger MB, Robson DN, Li JM, Keller PJ (2013). Whole-brain functional imaging at cellular resolution using light-sheet microscopy. Nat Methods.

[27] Portugues R, Feierstein CE, Engert F, Orger MB (2014). Whole-brain activity maps reveal stereotyped, distributed networks for visuomotor behavior. Neuron.

[28] Naumann EA, Fitzgerald JE, Dunn TW, Rihel J, Sompolinsky H, Engert F (2016). From whole-brain data to functional circuit models: the zebrafish optomotor response. Cell.

[29] Chen X, Mu Y, Hu Y, Kuan AT, Nikitchenko M, Randlett O, Chen AB, Gavornik JP, Sompolinsky H, Engert F (2018). Brain-wide organization of neuronal activity and convergent sensorimotor transformations in larval zebrafish. Neuron.

[30] Dunn TW, Gebhardt C, Naumann EA, Riegler C, Ahrens MB, Engert F, Del Bene F (2016). Neural circuits underlying visually evoked escapes in larval zebrafish. Neuron.

[31] Kawashima T, Zwart MF, Yang CT, Mensh BD, Ahrens MB (2016). The serotonergic system tracks the outcomes of actions to mediate short-term motor learning. Cell.

[32] Lin Q, Manley J, Helmreich M, Schlumm F, Li JM, Robson DN, Engert F, Schier A, Nöbauer T, Vaziri A (2020). Cerebellar neurodynamics predict decision timing and outcome on the single-trial level. Cell.

[33] Romano SA, Pietri T, Pérez-Schuster V, Jouary A, Haudrechy M, Sumbre G (2015). Spontaneous neuronal network dynamics reveal circuit’s functional adaptations for behavior. Neuron.

[34] Ponce-Alvarez A, Jouary A, Privat M, Deco G, Sumbre G (2018). Whole-brain neuronal activity displays crackling noise dynamics. Neuron.

[35] Avitan L, Pujic Z, Mölter J, Van De Poll M, Sun B, Teng H, et al. Spontaneous activity in the Zebrafish Tectum reorganizes over development and is influenced by visual experience. Curr Biol. 2017;27(16):2407–19.e2404. 10.1016/j.cub.2017.06.056 28781054

[36] Pietri T, Romano SA, Perez-Schuster V, Boulanger-Weill J, Candat V, Sumbre G (2017). The emergence of the spatial structure of tectal spontaneous activity is independent of visual inputs. Cell Rep.

[37] Deco G, Jirsa VK, McIntosh AR (2011). Emerging concepts for the dynamical organization of resting-state activity in the brain. Nat Rev Neurosci.

[38] Biswal B, Yetkin FZ, Haughton VM, Hyde JS (1995). Functional connectivity in the motor cortex of resting human brain using echo-planar MRI. Magn Reson Med.

[39] Raichle ME, MacLeod AM, Snyder AZ, Powers WJ, Gusnard DA, Shulman GL (2001). A default mode of brain function. Proc Natl Acad Sci U S A.

[40] Greicius M (2008). Resting-state functional connectivity in neuropsychiatric disorders. Curr Opin Neurol.

[41] Wu Y, Ghitani A, Christensen R, Santella A, Du Z, Rondeau G, Bao Z, Colón-Ramos D, Shroff H (2011). Inverted selective plane illumination microscopy (iSPIM) enables coupled cell identity lineaging and neurodevelopmental imaging in Caenorhabditis elegans. Proc Natl Acad Sci.

[42] Wilson SW, Houart C (2004). Early steps in the development of the forebrain. Dev Cell.

[43] Puelles L, Rubenstein JL (2003). Forebrain gene expression domains and the evolving prosomeric model. Trends Neurosci.

[44] Jernigan TL, Stiles J (2017). Construction of the human forebrain. Wiley Interdiscip Rev Cogn Sci.

[45] Tosches MA, Arendt D (2013). The bilaterian forebrain: an evolutionary chimaera. Curr Opin Neurobiol.

[46] Randlett O, Wee CL, Naumann EA, Nnaemeka O, Schoppik D, Fitzgerald JE, Portugues R, Lacoste AMB, Riegler C, Engert F (2015). Whole-brain activity mapping onto a zebrafish brain atlas. Nat Methods.

[47] Jia H, Rochefort NL, Chen X, Konnerth A (2011). In vivo two-photon imaging of sensory-evoked dendritic calcium signals in cortical neurons. Nat Protoc.

[48] Rohlfing T, Maurer CR (2003). Nonrigid image registration in shared-memory multiprocessor environments with application to brains, breasts, and bees. IEEE Trans Inform Technol Biomed.

[49] MacQueen JB (1967). Some methods for classification and analysis of multivariate observations. Proc 5-th Berkeley Symp Math Stat Prob.

[50] Watts DJ, Strogatz SH (1998). Collective dynamics of ‘small-world’ networks. Nature.

[51] Barabasi AL, Albert R (1999). Emergence of scaling in random networks. Science.

[52] Davison AC, Hinkley D, V. (1997). Bootstrap methods and their application.

[53] Pissanetzky S (1984). Sparse matrix technology-electronic edition.

[54] Calhoun VD, Sui J, Kiehl K, Turner J, Allen E, Pearlson G (2012). Exploring the psychosis functional connectome: aberrant intrinsic networks in schizophrenia and bipolar disorder. Front Psych.

[55] Hillman EM (2014). Coupling mechanism and significance of the BOLD signal: a status report. Annu Rev Neurosci.

[56] Lancaster JL, Woldorff MG, Parsons LM, Liotti M, Freitas CS, Rainey L, Kochunov PV, Nickerson D, Mikiten SA, Fox PT (2000). Automated Talairach atlas labels for functional brain mapping. Hum Brain Mapp.

[57] Raichle ME (2015). The brain's default mode network. Annu Rev Neurosci.

[58] Fox MD, Zhang D, Snyder AZ, Raichle ME (2009). The global signal and observed anticorrelated resting state brain networks. J Neurophysiol.

[59] Power JD, Plitt M, Laumann TO, Martin A (2017). Sources and implications of whole-brain fMRI signals in humans. Neuroimage.

[60] Fox MD, Raichle ME (2007). Spontaneous fluctuations in brain activity observed with functional magnetic resonance imaging. Nat Rev Neurosci.

[61] Di X, Kim EH, Huang CC, Tsai SJ, Lin CP, Biswal BB (2013). The influence of the amplitude of low-frequency fluctuations on resting-state functional connectivity. Front Hum Neurosci.

[62] Zang YF, He Y, Zhu CZ, Cao QJ, Sui MQ, Liang M, Tian LX, Jiang TZ, Wang YF (2007). Altered baseline brain activity in children with ADHD revealed by resting-state functional MRI. Brain Dev.

[63] Shulman RG, Rothman DL, Behar KL, Hyder F (2004). Energetic basis of brain activity: implications for neuroimaging. Trends Neurosci.

[64] Kumar A, Wu Y, Christensen R, Chandris P, Gandler W, McCreedy E, Bokinsky A, Colón-Ramos DA, Bao Z, McAuliffe M (2014). Dual-view plane illumination microscopy for rapid and spatially isotropic imaging. Nat Protoc.

[65] Thévenaz P, Ruttimann UE, Unser M (1998). A pyramid approach to subpixel registration based on intensity. IEEE Trans Image Process.

[66] van der Walt S, Schonberger JL, Nunez-Iglesias J, Boulogne F, Warner JD, Yager N, Gouillart E, Yu T (2014). Scikit-image: image processing in Python. Peer J.

[67] Churchill NW, Oder A, Abdi H, Tam F, Lee W, Thomas C, Ween JE, Graham SJ, Strother SC (2012). Optimizing preprocessing and analysis pipelines for single-subject fMRI. I. Standard temporal motion and physiological noise correction methods. Hum Brain Mapp.

[68] Griffanti L, Douaud G, Bijsterbosch J, Evangelisti S, Alfaro-Almagro F, Glasser MF, Duff EP, Fitzgibbon S, Westphal R, Carone D (2017). Hand classification of fMRI ICA noise components. Neuroimage.

[69] Behzadi Y, Restom K, Liau J, Liu TT (2007). A component based noise correction method (CompCor) for BOLD and perfusion based fMRI. Neuroimage.

[70] Oliphant TE (2006). NumPy library of Python programming language, a guide to NumPy.

[71] Pedregosa F, Varoquaux G, Gramfort A, Michel V, Thirion B, Grisel O, Blondel M, Prettenhofer P, Weiss R, Dubourg V (2011). Scikit-learn: machine learning in python. JMLR.

[72] CLeveland WS (1979). Robust locally weighted regression and smoothing scatterplo. J Am Stat Assoc.

