# Supplementary material for: High activity and high functional connectivity are mutually exclusive in resting state zebrafish and human brains
Source: BMC Biol. 2022 Apr 11;20:84. doi: 10.1186/s12915-022-01286-3 (PMC8996543; doi:10.1186/s12915-022-01286-3)
Supplement: Supplementary file 1 — Additional file 1. Light-sheet imaging and image processing generate large-scale single neuron activity data. [file 12915_2022_1286_MOESM1_ESM.pdf]

Supplementary Materials

Additional File 1

Light-sheet imaging and image processing generate large-scale single neuron activity data

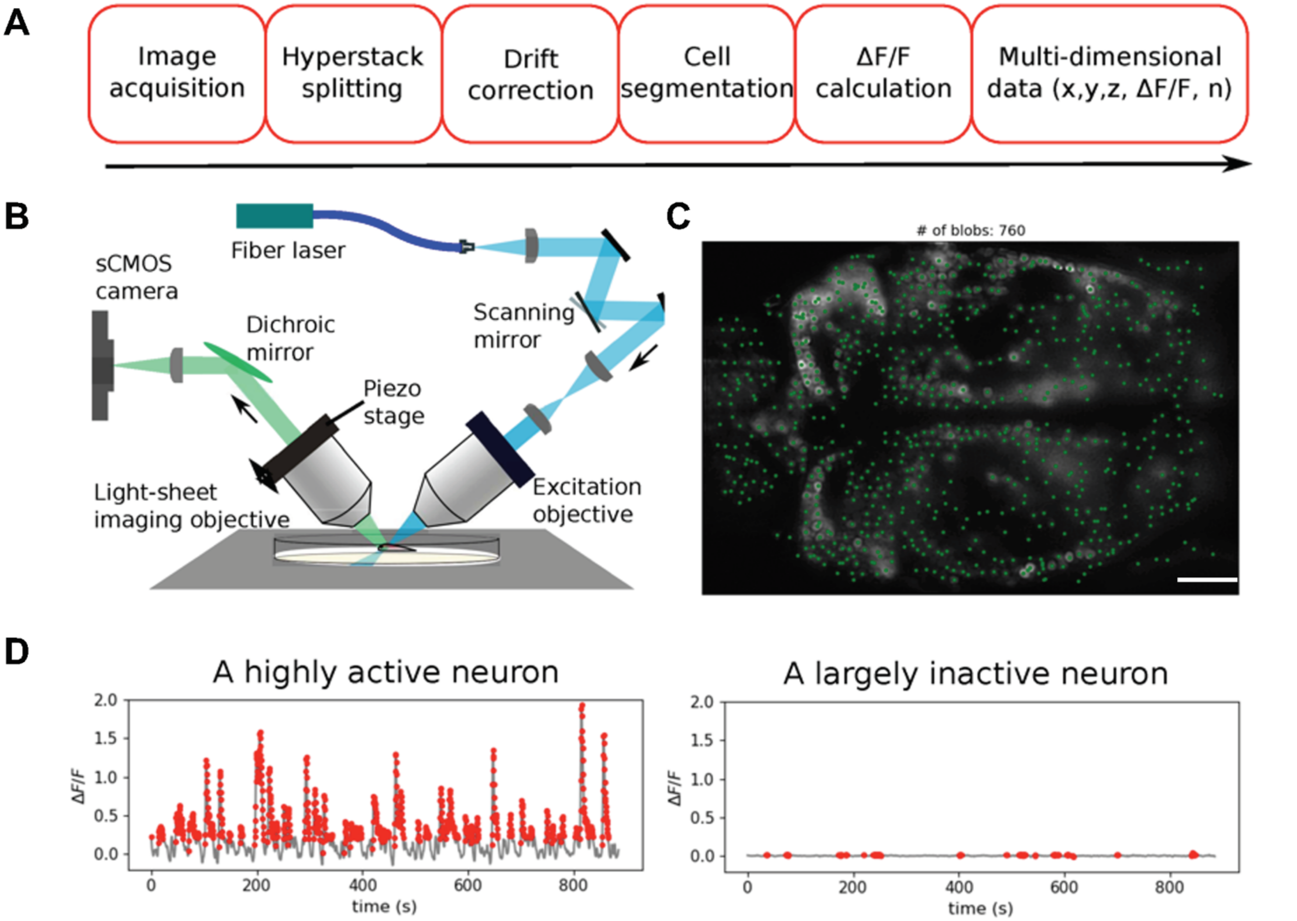

E

Subject	1	2	3	4	5	6	7	8	9
#Cells	15705	13947	17111	13653	17100	14373	15126	12315	13791

Additional File 1. Light-sheet imaging and image processing generate large-scale single neuron activity data. A, a schematic showing the overall workflow of image acquisition and pre-processing pipeline resulting in a multi-dimensional dataset. The df/f, 3d coordinate, and anatomical mask was assigned to each detected cell. **B,** a schematic showing the setup of the iSPIM *in vivo* calcium imaging system. **C,** a representative image of 6 dpf larval zebrafish forebrain showing detected cells (blobs) (n=760 cells detected). **D,** An example of a highly active (left) and a largely inactive (right) neurons. Red dots show peaks above the baselines from each ΔF/F time trace, which is based on the Bayesian inference of two-dimensional distribution of adjacent ΔF/F values (See Methods)**. E,** Number of detected cells in each of the 9 subjects used in this study. scale bar, 25 um; dpf, days post fertilization. The number of replicates used is 9.
