# Supplementary material for: High activity and high functional connectivity are mutually exclusive in resting state zebrafish and human brains
Source: BMC Biol. 2022 Apr 11;20:84. doi: 10.1186/s12915-022-01286-3 (PMC8996543; doi:10.1186/s12915-022-01286-3)
Supplement: Supplementary file 2 — Additional file 2. Brain registration enables comparison of single-neuron activity across different individuals. [file 12915_2022_1286_MOESM2_ESM.pdf]

Additional File 2

Brain registration enables comparison of single-neuron activity across different individuals

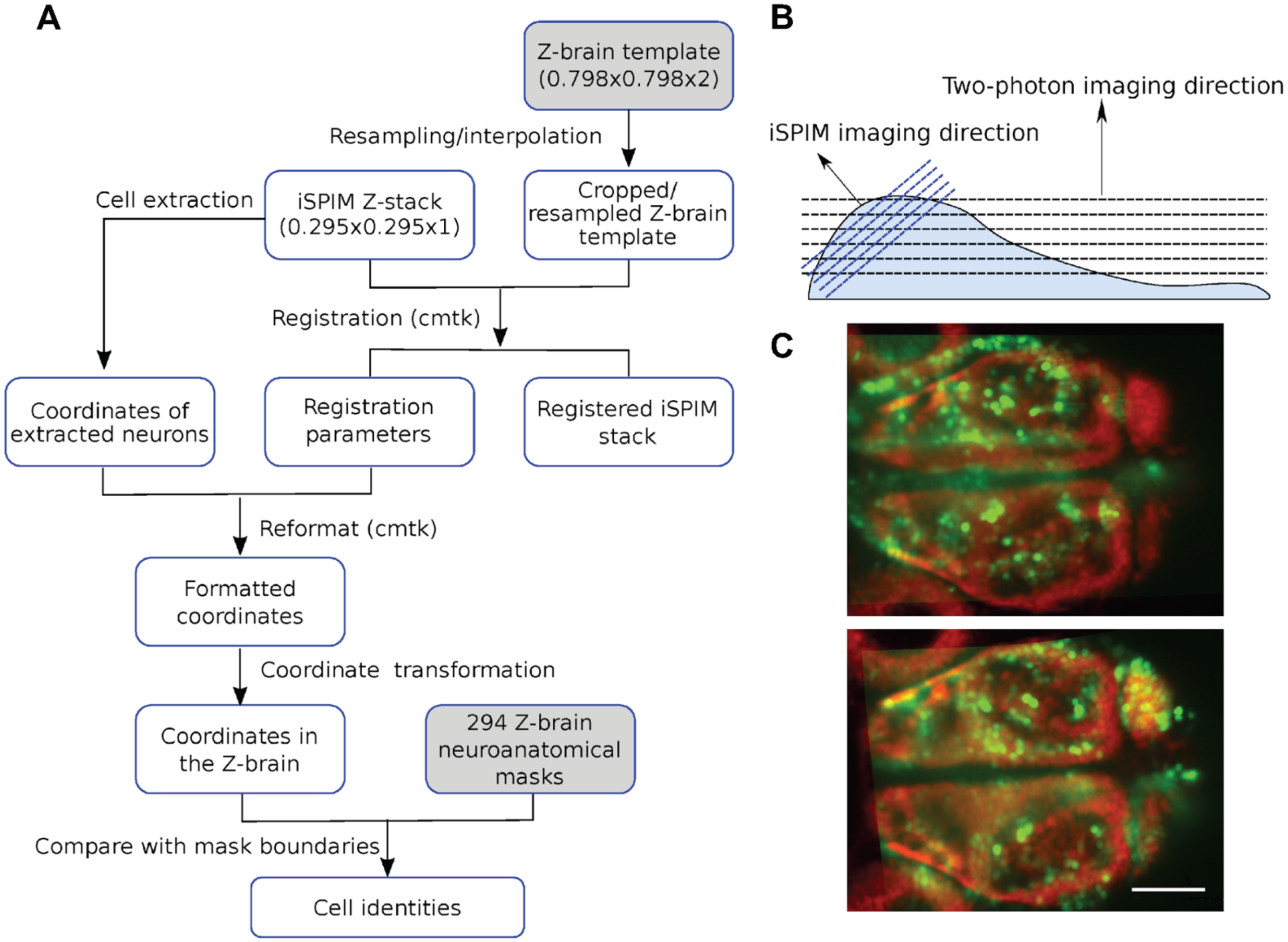

Additional File 2. Brain registration enables comparison of single-neuron activity across different individuals. A, a schematic showing the workflow of image registration and anatomical labeling. **B,** a schematic showing the imaging direction in the iSPIM (blue) and the 2-photon system (black), respectively. Stacks acquired from the former need to be rotated during image registration to the Z-brain atlas, which is acquired via 2-photon microscopy. **C**, Example image slice of dorsal forebrain, before (top) and after (bottom) registration. Red: 2-photon image of HuC-H2B-RFP labeled forebrain template in the Z-brain atlas, cropped and resampled in the iSPIM imaging direction. Green: HuC-H2B-GCAMP6s labeling. Scale bar, 50 um.
