# Supplementary material for: High activity and high functional connectivity are mutually exclusive in resting state zebrafish and human brains
Source: BMC Biol. 2022 Apr 11;20:84. doi: 10.1186/s12915-022-01286-3 (PMC8996543; doi:10.1186/s12915-022-01286-3)
Supplement: Supplementary file 3 — Additional file 3. Evaluation of the accuracy and representation of anatomical label assignment. [file 12915_2022_1286_MOESM3_ESM.pdf]

Additional File 3

A

Evaluation of the accuracy of anatomical labeling in an example subject

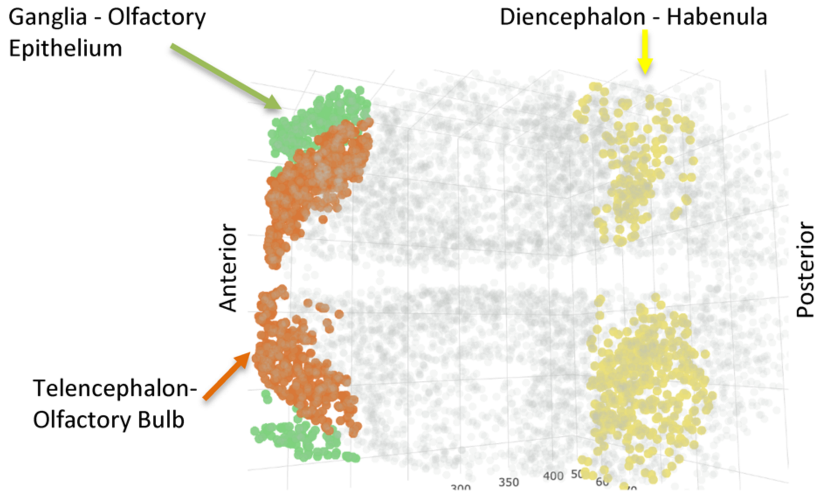

Numbers of ROIs detected in anatomical labels of an example subject

B

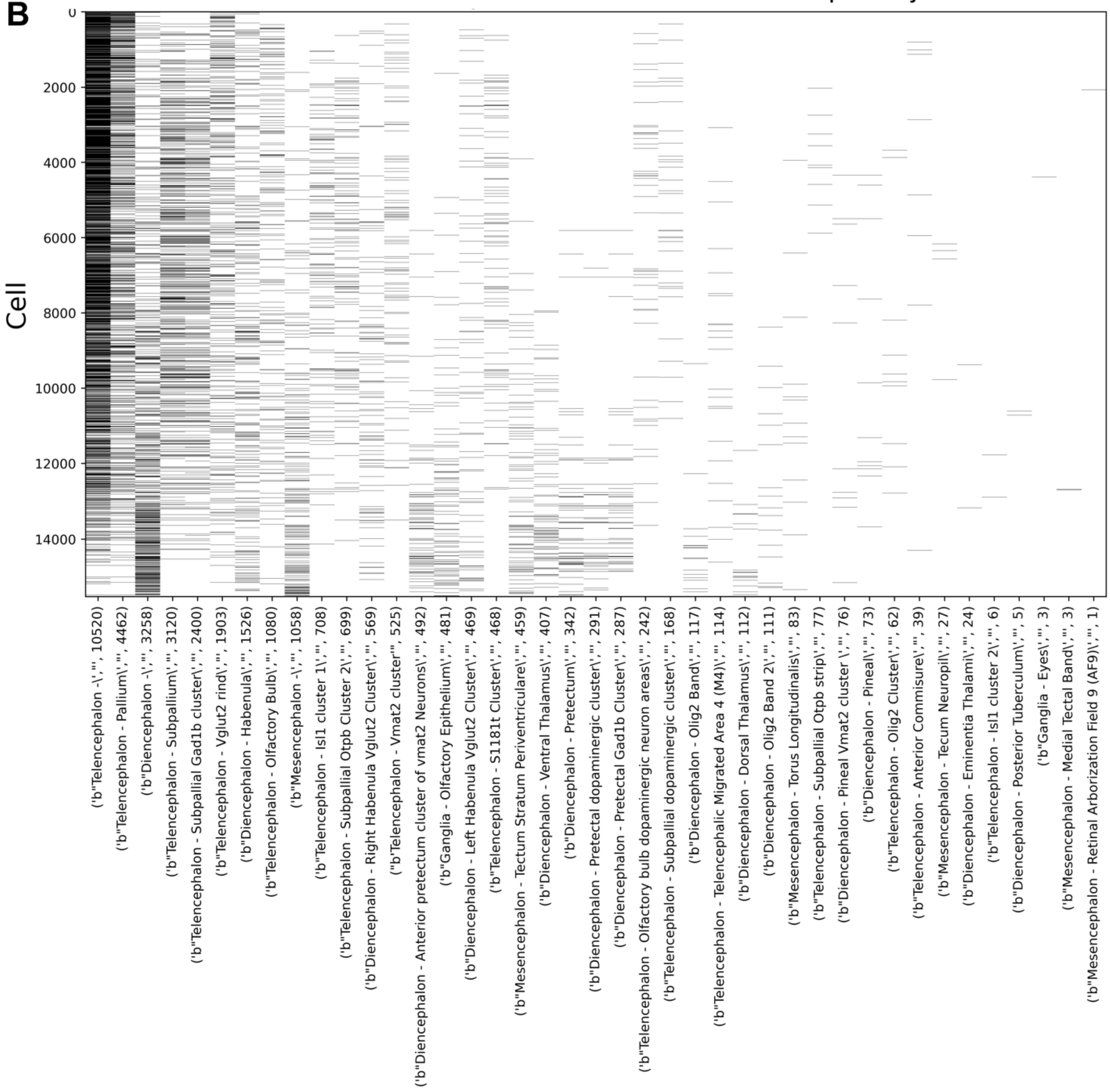

Additional File 3. Evaluation of the accuracy and representation of anatomical label assignment. A, 2-D visualization of recorded ROIs (individual neurons) in several representative anatomic regions of an example subject. **B,** a binary heatmap showing the number of cells detected in defined neuroanatomical labels.
