# Supplementary material for: High activity and high functional connectivity are mutually exclusive in resting state zebrafish and human brains
Source: BMC Biol. 2022 Apr 11;20:84. doi: 10.1186/s12915-022-01286-3 (PMC8996543; doi:10.1186/s12915-022-01286-3)
Supplement: Supplementary file 4 — Additional file 4. Identification of optimal threshold values to uncover significant functional connections in larval zebrafish calcium imaging data. [file 12915_2022_1286_MOESM4_ESM.pdf]

Additional File 4

Identification of optimal thresholding values to uncover potentially genuine connections

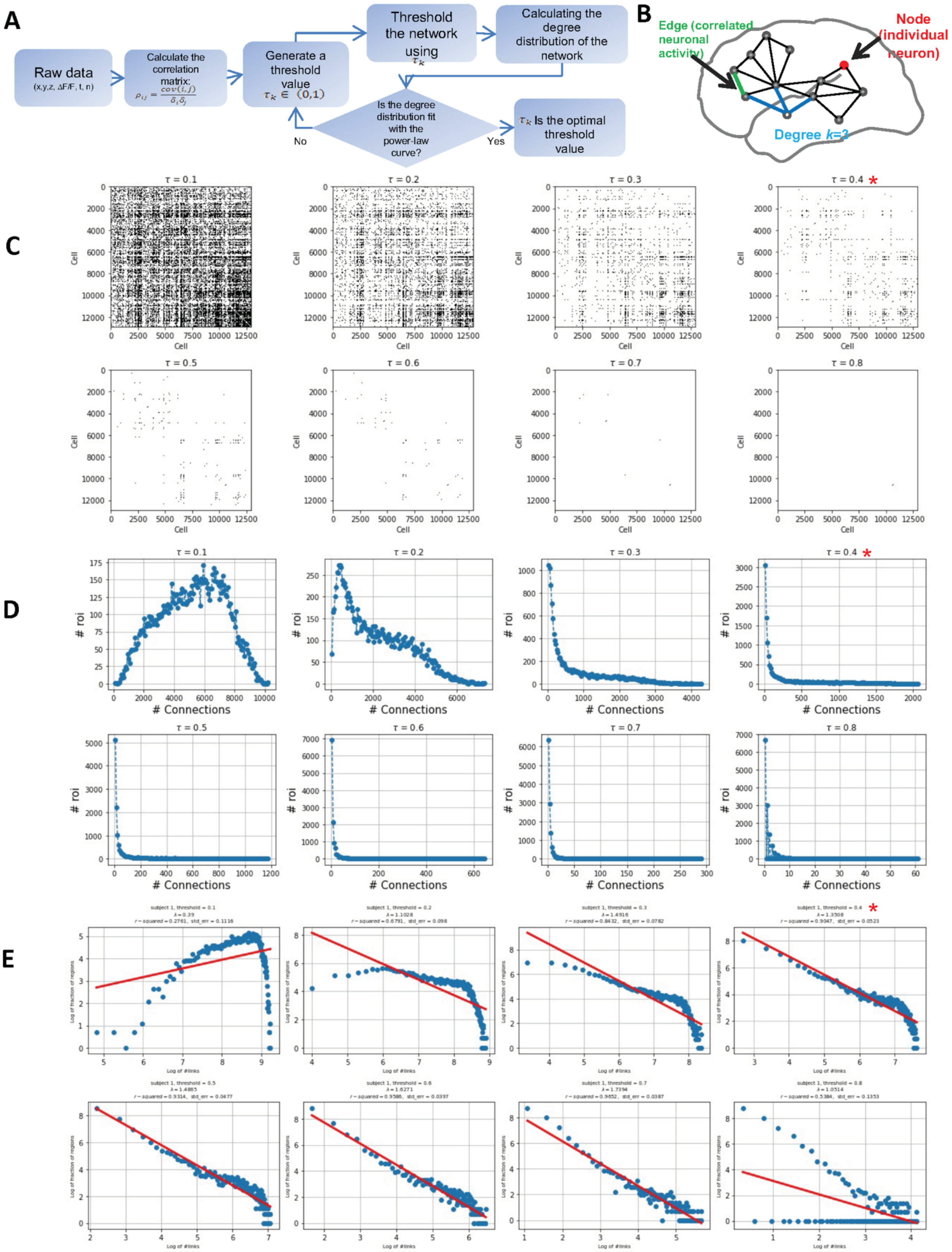

Additional File 4. Identification of optimal threshold values to uncover significant functional connections in larval zebrafish calcium imaging data. A, a schematic showing the workflow of calculating functional connectivity for each ROI. **B,** a schematic of complex brain network, in which individual neurons are considered as nodes and the statistically significant relationships between each pair of nodes are known as edges. The number of edges each node has is called the degree. **C-E,** graphs for an example subject. (**C**) correlation matrices of different sparsity using different threshold values as indicated. Connections below the thresholding values are removed. **D**, Graphs showing degree distributions calculated from connectivity matrices as shown in c. (**E)** Graphs showing the approximation of line on the log-log scale to find the optimal threshold value at which data follow a power law. The optimal threshold value is 0.4 with a highest r^2 (0.97) (marked with a red asterisk).
