# Supplementary material for: High activity and high functional connectivity are mutually exclusive in resting state zebrafish and human brains
Source: BMC Biol. 2022 Apr 11;20:84. doi: 10.1186/s12915-022-01286-3 (PMC8996543; doi:10.1186/s12915-022-01286-3)
Supplement: Supplementary file 5 — Additional file 5. Analysis of the shuffled larval zebrafish calcium imaging data does not show power law distribution at any thresholding values. [file 12915_2022_1286_MOESM5_ESM.pdf]

Additional File 5

Apply power law calculations to the Shuffled data

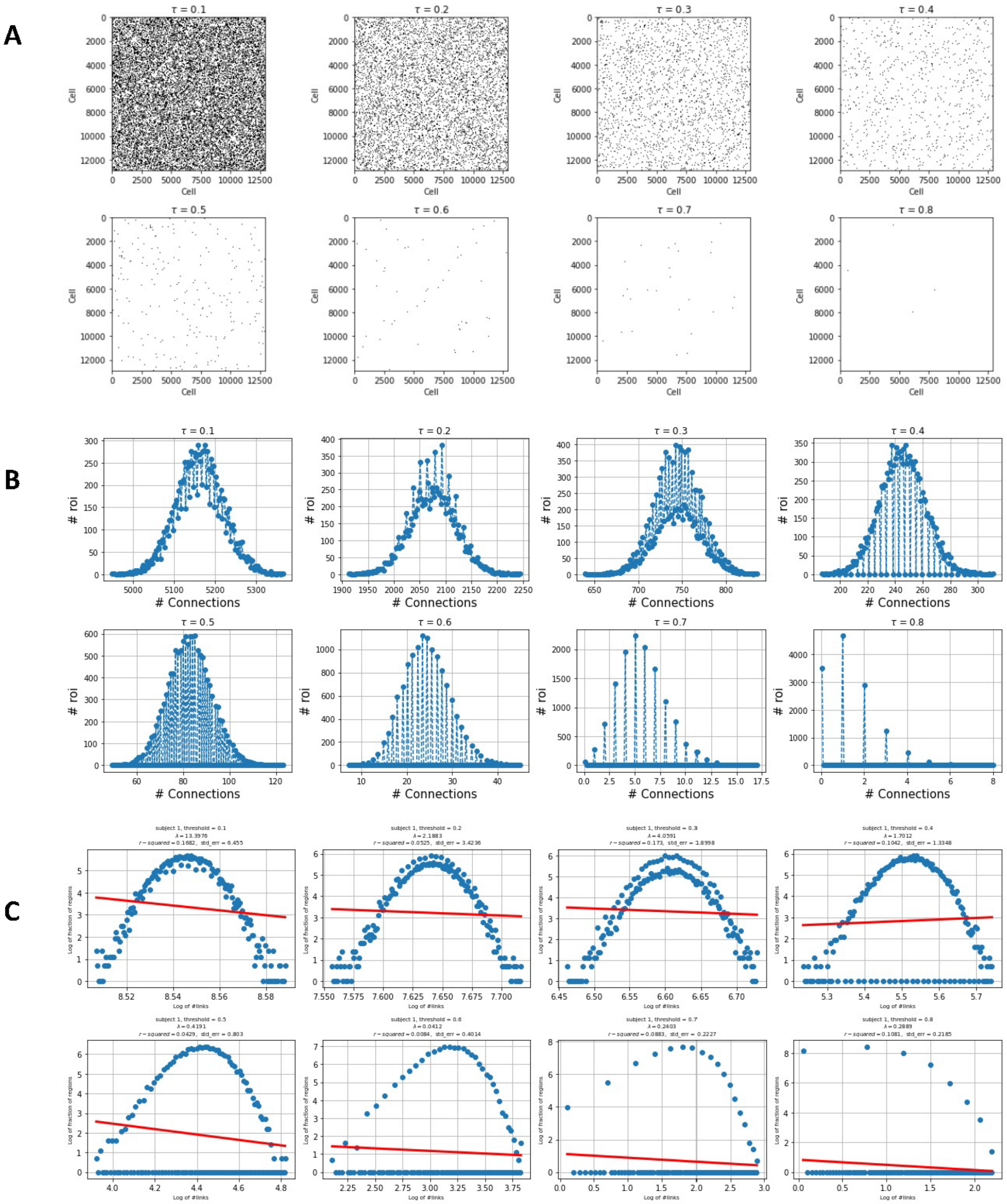

Additional File 5. Analysis of the shuffled larval zebrafish calcium imaging data does not show power law distribution at any thresholding values. **A**, correlation matrices of different sparsity using different threshold values as indicated. Connections below the thresholding values are removed. **B**, Graphs showing degree distributions calculated from connectivity matrices as shown in A. **C**, graphs showing the approximation of line on the log-log scale. No power law distribution is observed at any thresholding value.
