# Supplementary material for: High activity and high functional connectivity are mutually exclusive in resting state zebrafish and human brains
Source: BMC Biol. 2022 Apr 11;20:84. doi: 10.1186/s12915-022-01286-3 (PMC8996543; doi:10.1186/s12915-022-01286-3)
Supplement: Supplementary file 6 — Additional file 6. Validation of detected functional connections in the larval zebrafish forebrain. [file 12915_2022_1286_MOESM6_ESM.pdf]

Additional File 6

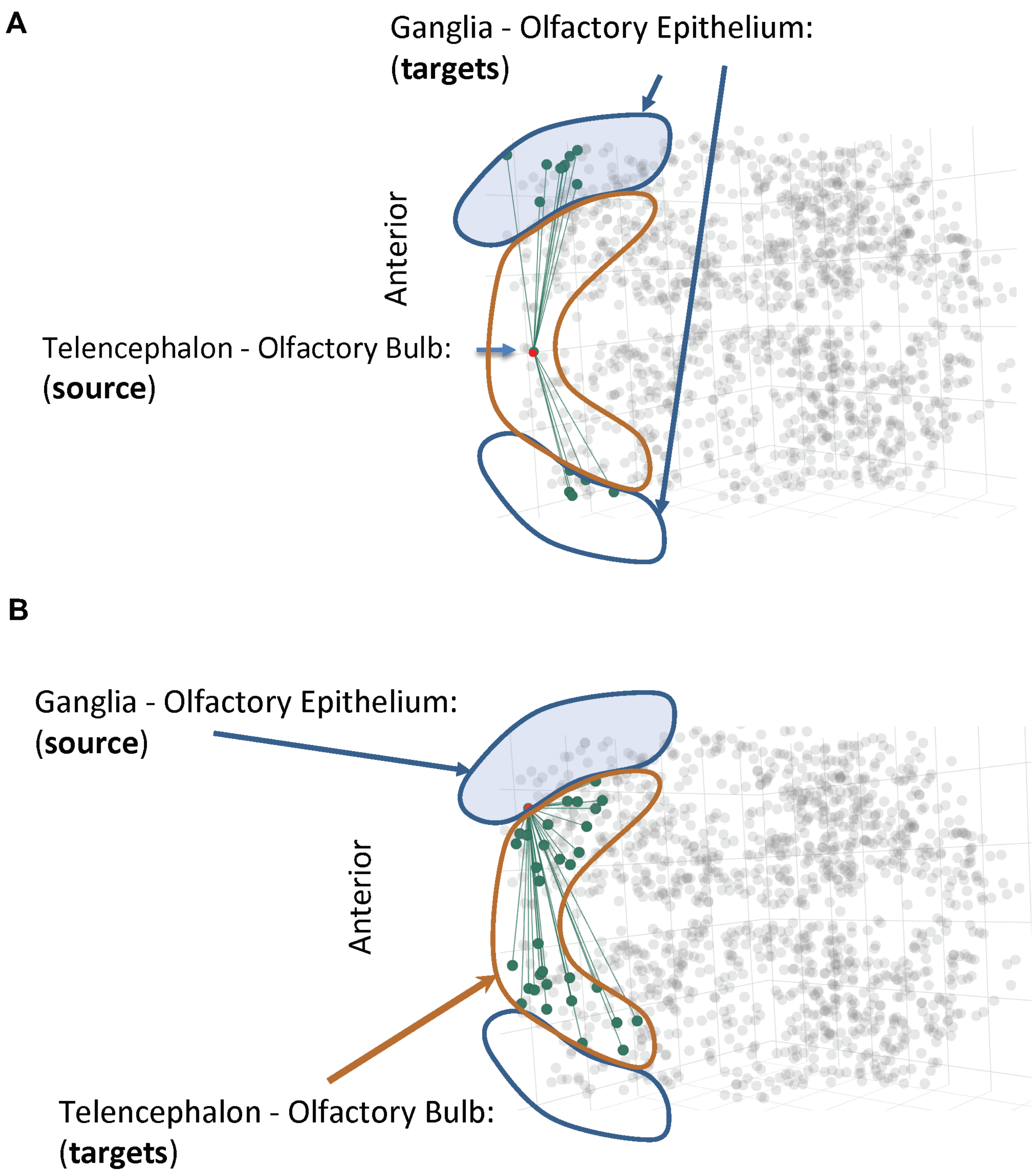

Additional File 6. Validation of detected functional connections in the larval zebrafish forebrain. A, a source cell in the Telencephalon Olfactory blub is detected to have functional connections with cells in the olfactory epithelium. **B**, a source cell in the Ganglia - Olfactory Epithelium is detected to make functional connections with cells in the Telencephalon Olfactory blub. Olfactory epithelium is known to be connected with Olfactory bulb, thus validating our method of detecting functional connectivity.
