# Supplementary material for: High activity and high functional connectivity are mutually exclusive in resting state zebrafish and human brains
Source: BMC Biol. 2022 Apr 11;20:84. doi: 10.1186/s12915-022-01286-3 (PMC8996543; doi:10.1186/s12915-022-01286-3)
Supplement: Supplementary file 7 — Additional file 7. Highly active and highly connected neuronal populations are largely non-overlapping when analyzed using 5 clusters. [file 12915_2022_1286_MOESM7_ESM.pdf]

Additional File 7. Highly active and highly connected neuronal populations are largely non-overlapping when analyzed using 5 clusters.

A

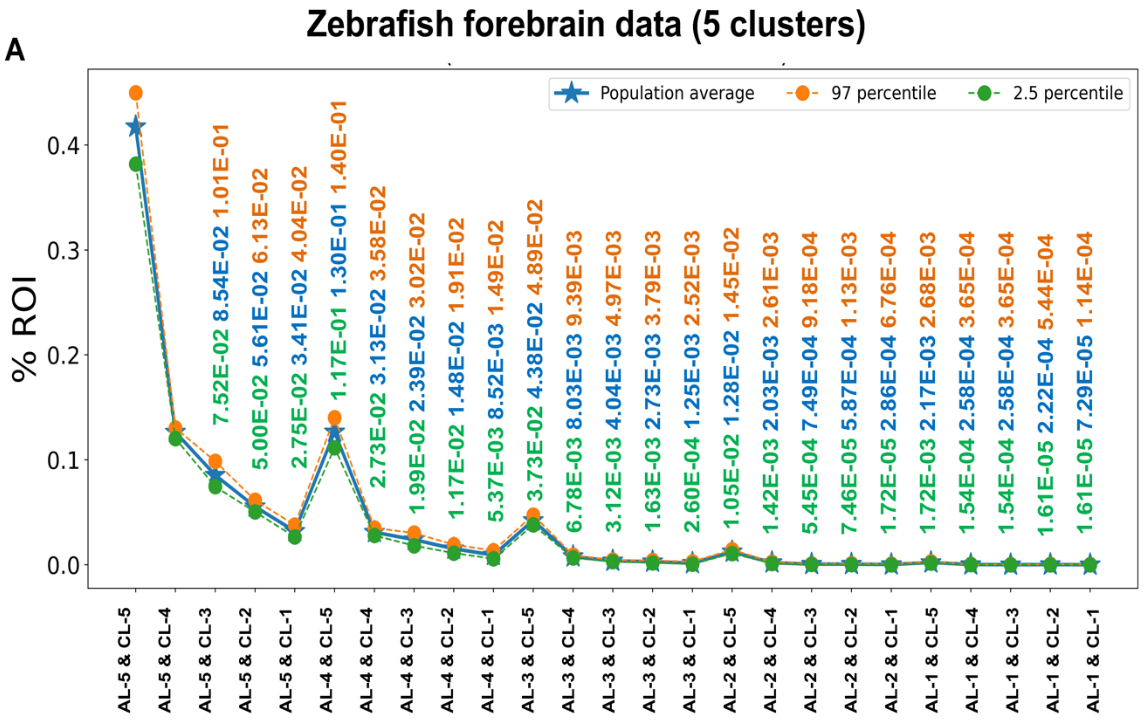

B

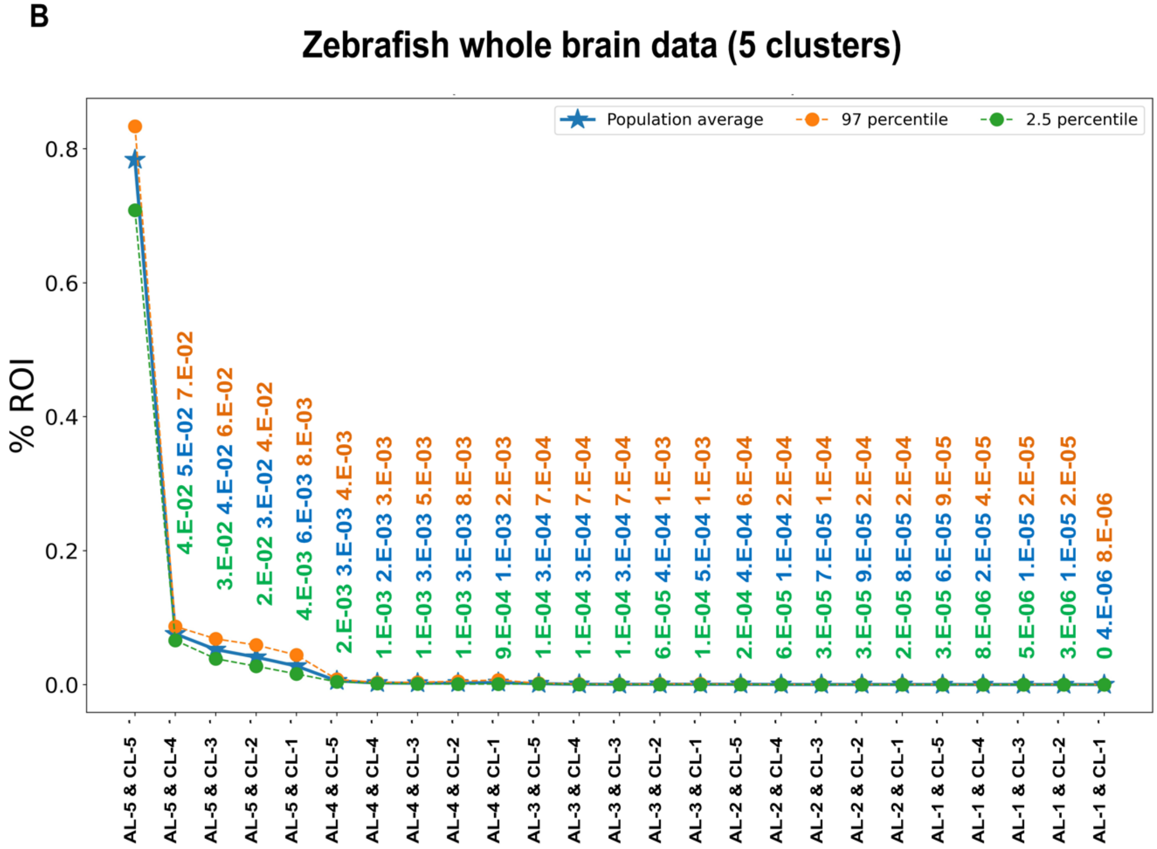

Additional File 7. Highly active and highly connected neuronal populations are largely non-overlapping when analyzed using 5 clusters. The population distribution curve of all neurons with different levels of activity and functional connectivity categorized into 5 clusters using k-means. **A**, zebrafish forebrain data. **B**, zebrafish whole brain data. Similar distributions were observed as the analysis done with 3 clusters using k-means.
