# Supplementary material for: High activity and high functional connectivity are mutually exclusive in resting state zebrafish and human brains
Source: BMC Biol. 2022 Apr 11;20:84. doi: 10.1186/s12915-022-01286-3 (PMC8996543; doi:10.1186/s12915-022-01286-3)
Supplement: Supplementary file 8 — Additional file 8. A flowchart showing the human brain data preprocessing and analysis pipeline. [file 12915_2022_1286_MOESM8_ESM.pdf]

Additional File 8. Human brain data preprocessing and analysis pipeline

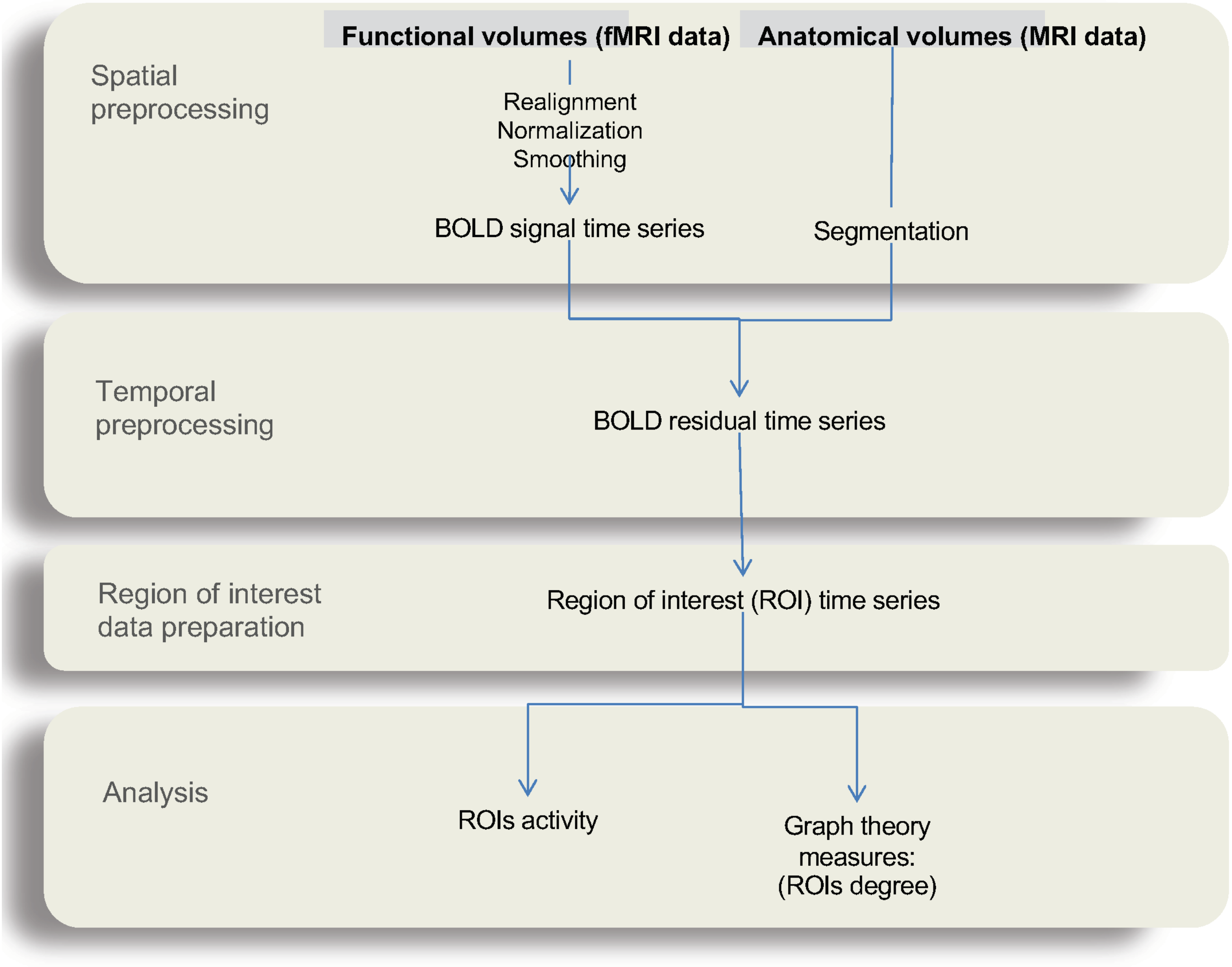

Additional File 8. A flowchart showing the human brain data preprocessing and analysis pipeline.
