# Supplementary material for: High activity and high functional connectivity are mutually exclusive in resting state zebrafish and human brains
Source: BMC Biol. 2022 Apr 11;20:84. doi: 10.1186/s12915-022-01286-3 (PMC8996543; doi:10.1186/s12915-022-01286-3)
Supplement: Supplementary file 9 — Additional file 9. Characterization of activity in the human brain. [file 12915_2022_1286_MOESM9_ESM.pdf]

Additional File 9. Characterization of activity in the human brain

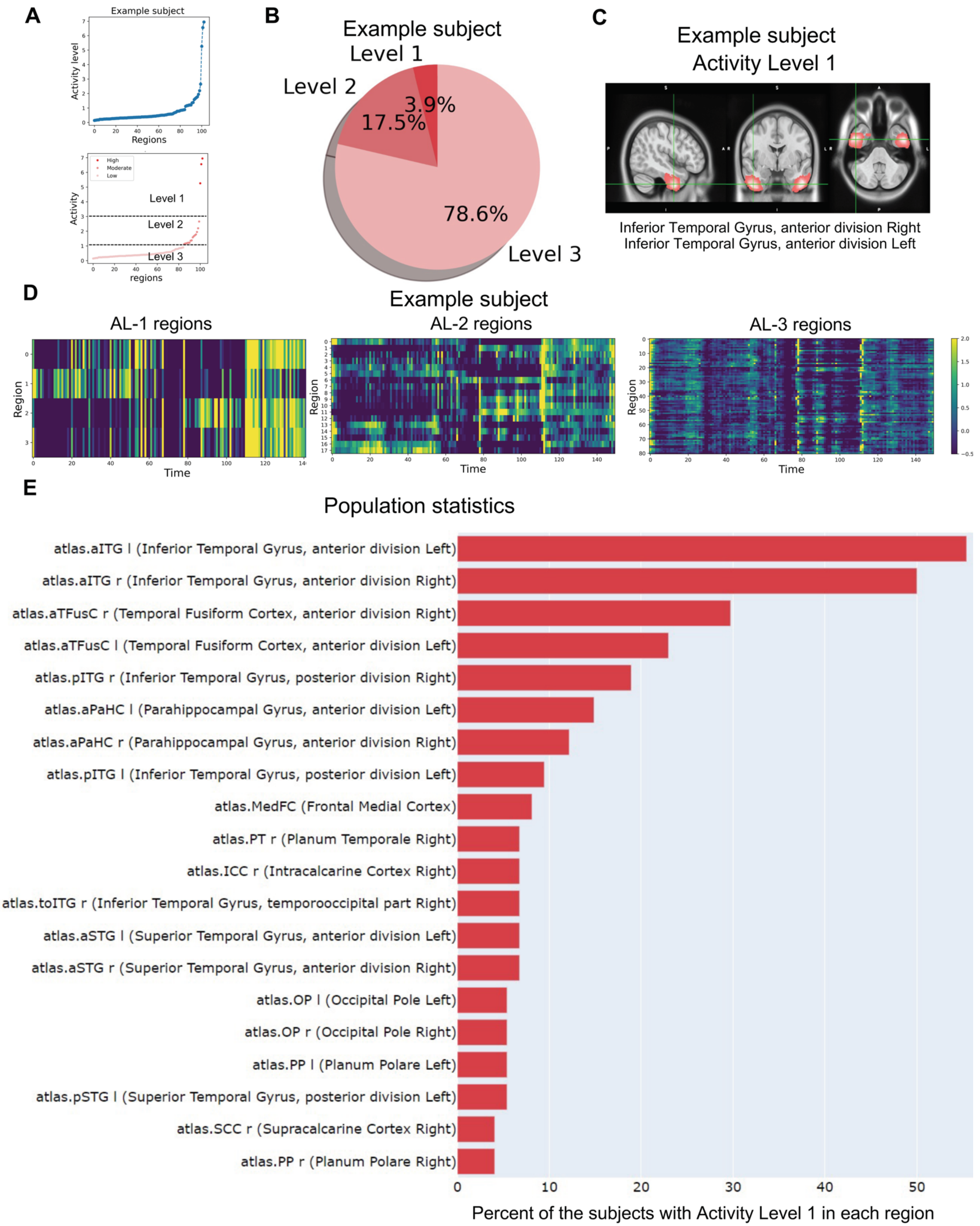

Additional File 9. Characterization of activity in the human brain. A, the sorted (top) and the clustered activity levels using k-means algorithm (bottom). **B**, percentage of brain regions belonging to each activity category for an example subject. **C,** highly active brain regions (e.g., Inferior Temporal Gyrus anterior division) in an example subject, which is shared across more than 45% of the subjects. **D,** the heatmap of the Activity Level 1 (left) and Activity Level 3 brain regions (right). **E,** Bar graph showing the top 20 brain regions in Activity Level 1 and percent of subjects classified to have Level 1 activity for each brain region. The number of replicates used is 74.
