# Supplementary material for: High activity and high functional connectivity are mutually exclusive in resting state zebrafish and human brains
Source: BMC Biol. 2022 Apr 11;20:84. doi: 10.1186/s12915-022-01286-3 (PMC8996543; doi:10.1186/s12915-022-01286-3)
Supplement: Supplementary file 10 — Additional file 10. Characterization of the connectivity in the human brain. [file 12915_2022_1286_MOESM10_ESM.pdf]

Additional File 10. Characterization of connectivity in the human brain

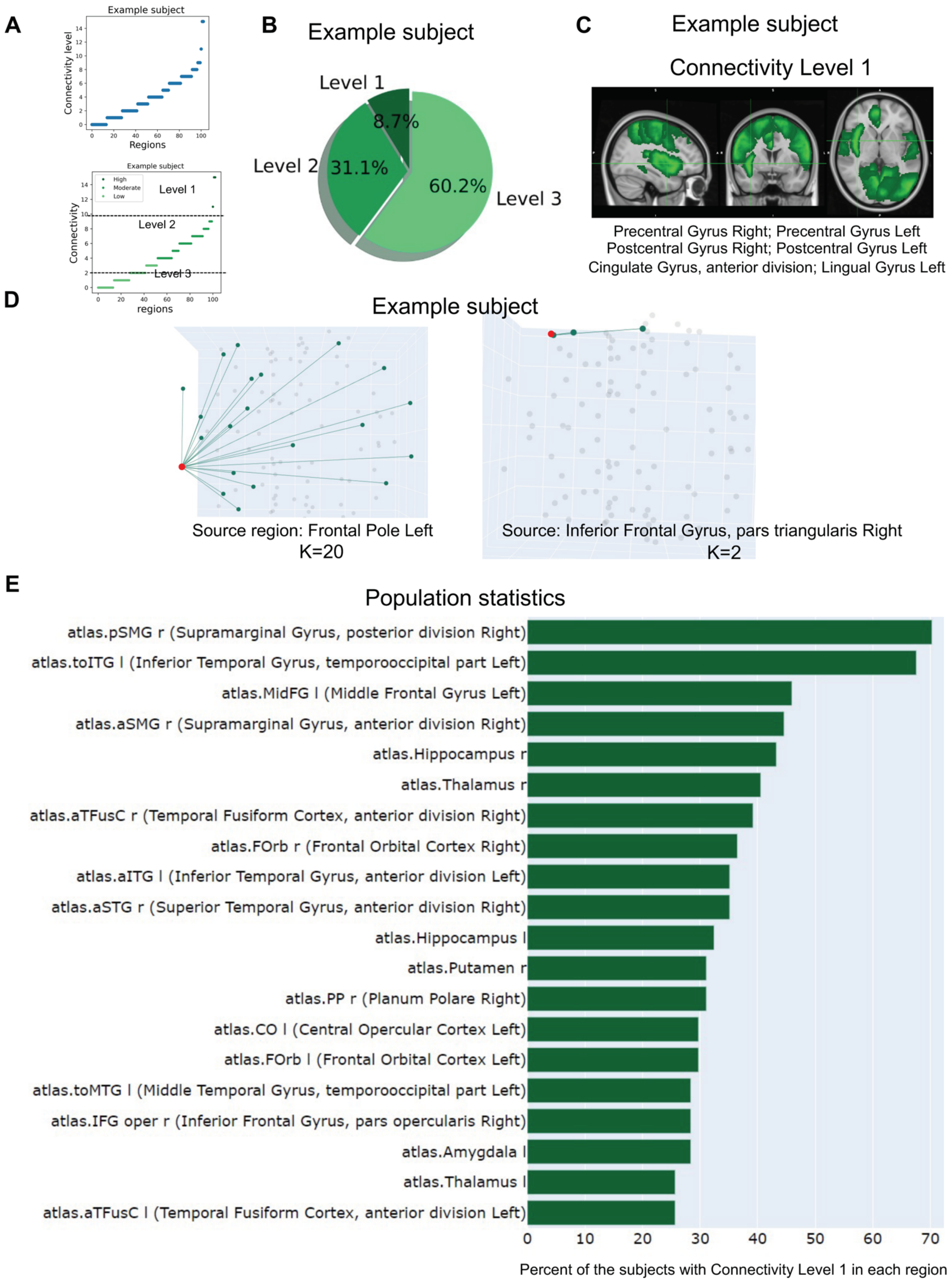

Additional File 10. Characterization of the connectivity in the human brain. A, the sorted (top) and the clustered connectivity levels using k-means algorithm (bottom). **B,** percentage of brain regions in each connectivity category for an example subject. **C,** highly connected brain regions that are shared across more than 45% of the subjects. **D,** the connectivity of two example brain regions in the Connectivity Level 1 (left) and 3 (right) categories of an example subject. **E,** Percent of the subjects for each brain region with the connectivity level 1. The number of replicates used is 74.
