# Supplementary material for: High activity and high functional connectivity are mutually exclusive in resting state zebrafish and human brains
Source: BMC Biol. 2022 Apr 11;20:84. doi: 10.1186/s12915-022-01286-3 (PMC8996543; doi:10.1186/s12915-022-01286-3)
Supplement: Supplementary file 11 — Additional file 11. Activity-connectivity relationship of example human data at different threshold levels for the connectivity matrix. [file 12915_2022_1286_MOESM11_ESM.pdf]

Additional File 11. Activity-connectivity relationship of example human data at different threshold levels for the connectivity matrix

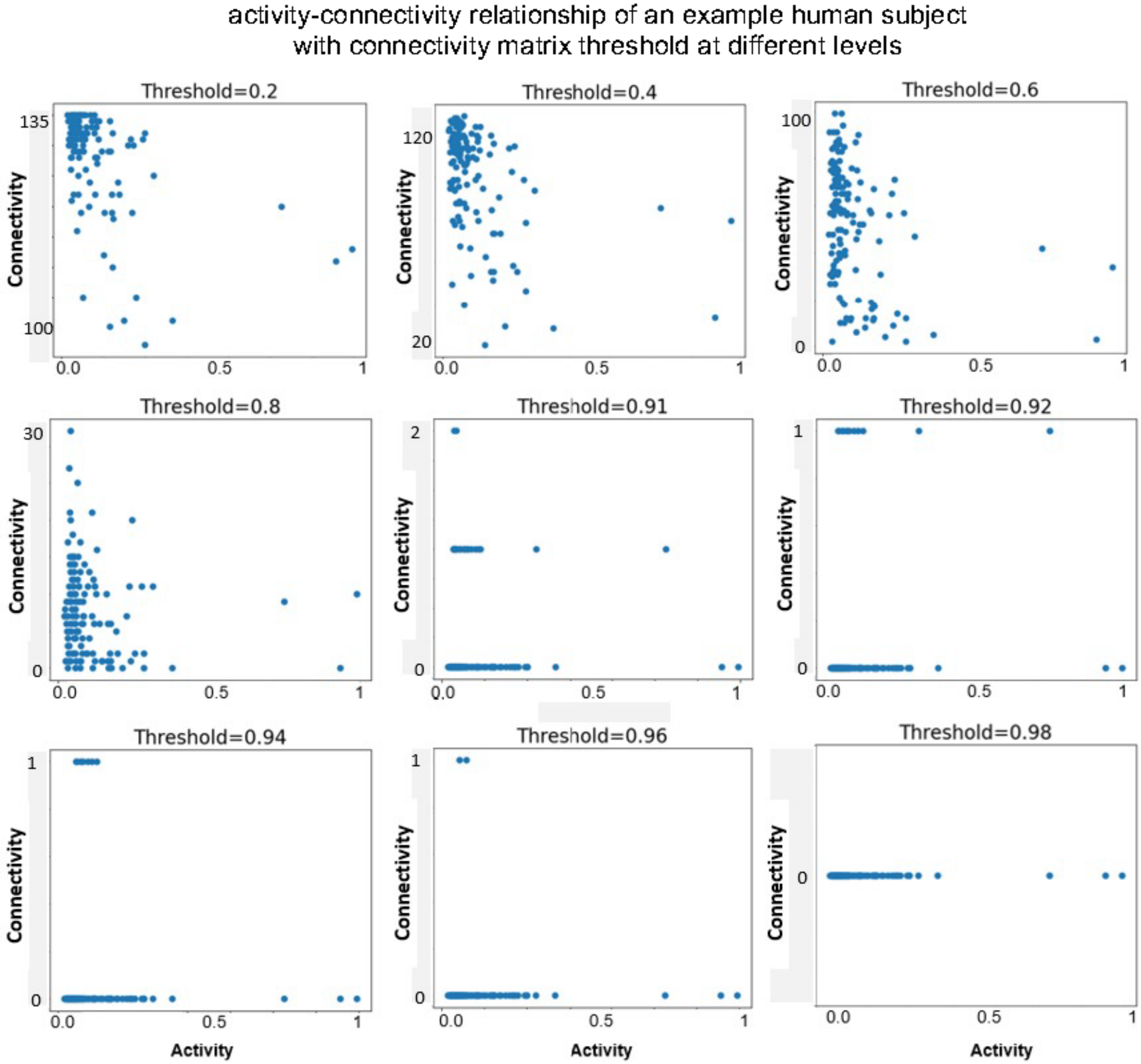

Additional File 11. Activity-connectivity relationship of example human data at different threshold levels for the connectivity matrix. Graphs showing activity-connectivity relationship. At thresholds much lower than the optimal threshold, ROIs with low activity were found to have very high connectivity, due to possible noise. At thresholds much higher than the optimal threshold, the connections among ROIs are gradually lost.
