# Supplementary material for: High activity and high functional connectivity are mutually exclusive in resting state zebrafish and human brains
Source: BMC Biol. 2022 Apr 11;20:84. doi: 10.1186/s12915-022-01286-3 (PMC8996543; doi:10.1186/s12915-022-01286-3)
Supplement: Supplementary file 12 — Additional file 12. The mutually exclusive relationship between high activity and high functional connectivity is observed when deriving functional connectivity without using a threshold. [file 12915_2022_1286_MOESM12_ESM.pdf]

Additional File 12: The mutually exclusive relationship between high activity and high functional connectivity is observed when deriving functional connectivity without using a threshold

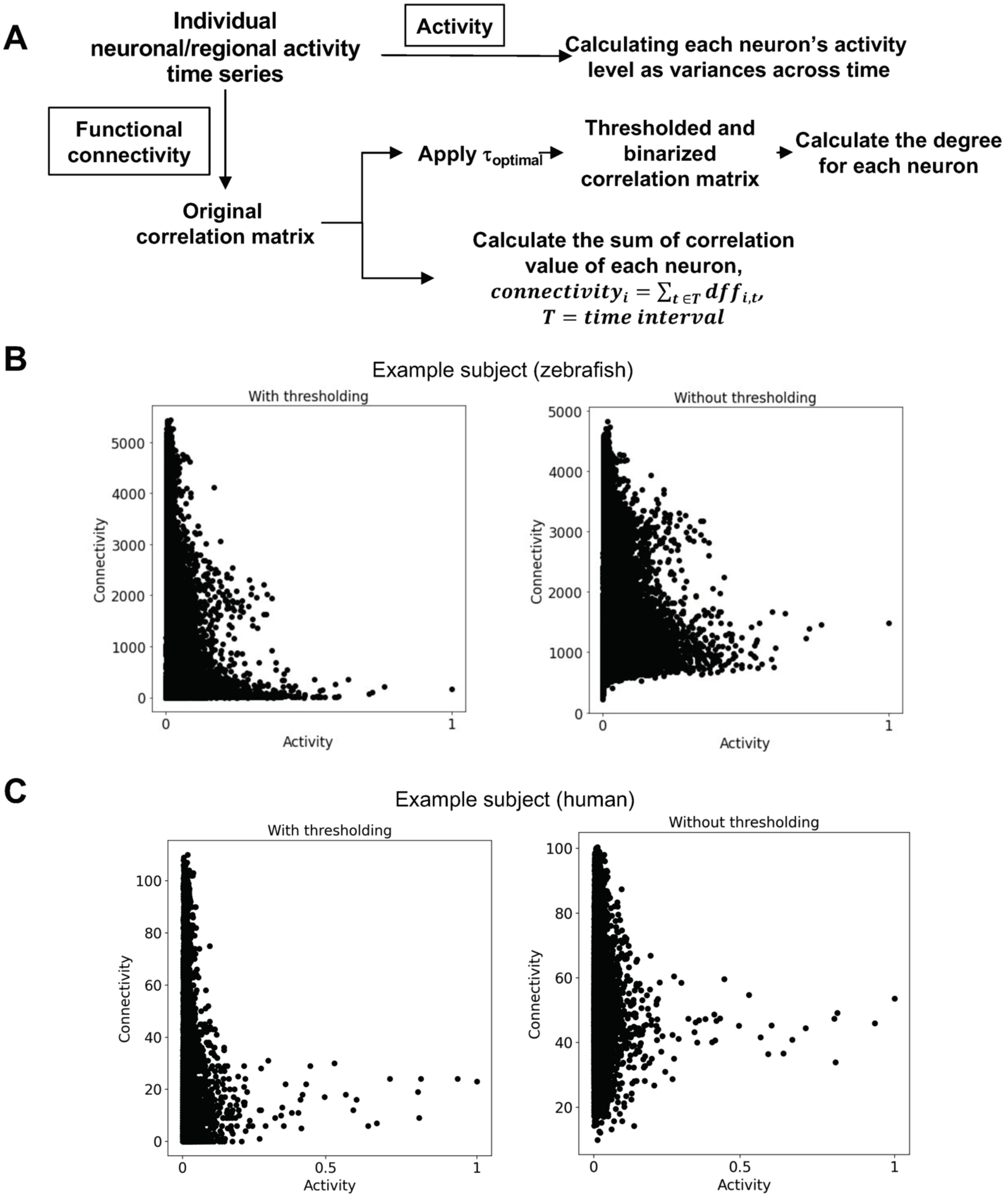

Additional File 12. The mutually exclusive relationship between high activity and high functional connectivity is observed when deriving functional connectivity without using a threshold. **A**, Flowchart showing the methods of calculating ROIs' activity and functional connectivity (with or without a thresholding procedure). **B**, graphs showing the activity-connectivity relationship with (left) and without (right) a thresholding procedure in a zebrafish forebrain example subject. **C**, graphs showing the activity-connectivity relationship with (left) and without (right) a thresholding procedure in a human example subject.
