# Supplementary material for: High activity and high functional connectivity are mutually exclusive in resting state zebrafish and human brains
Source: BMC Biol. 2022 Apr 11;20:84. doi: 10.1186/s12915-022-01286-3 (PMC8996543; doi:10.1186/s12915-022-01286-3)
Supplement: Supplementary file 13 — Additional file 13. Relationship between activity and connectivity in the shuffled or noise-added human data or simulated data. [file 12915_2022_1286_MOESM13_ESM.pdf]

Additional File 13: Relationship between activity and connectivity in the shuffled or noise-added human data or simulated data

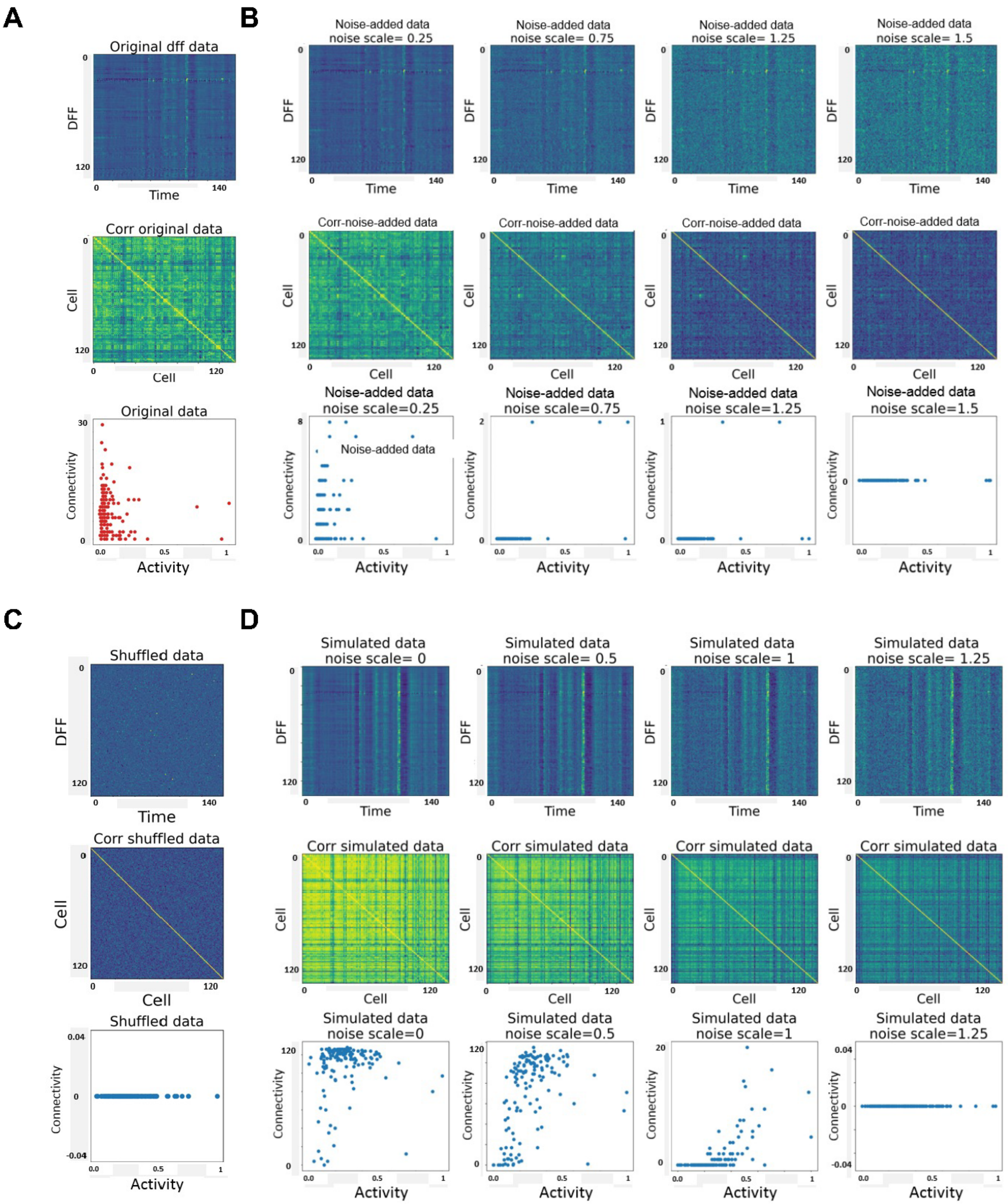

87

Additional File 13. Relationship between activity and connectivity in the shuffled or noise-added human data or simulated data. Different datasets with ROI activity time series (top), ROI-wise correlation matrix (middle), and the graphed functional connectivity and activity relationship (bottom). **A**, Original data of an example subject. **B**, Different levels of noise were added to the original data, resulting in the loss of the activity-connectivity relationship observed in the original brain data. **C**, ROI activity time series of the original data were shuffled in both space and time. The activity-connectivity relationship observed in the original brain data was lost. **D**, a simulated dataset shows the activity-connectivity relationship that is distinct to the brain data and is also sensitive to the levels of noise.
